# Optimal Balance of the Striatal Medium Spiny Neuron Network

**DOI:** 10.1371/journal.pcbi.1002954

**Published:** 2013-04-11

**Authors:** Adam Ponzi, Jeffery R. Wickens

**Affiliations:** Neurobiology Research Unit, Okinawa Institute of Science and Technology (OIST), Okinawa, Japan; Université Paris Descartes, Centre National de la Recherche Scientifique, France

## Abstract

Slowly varying activity in the striatum, the main Basal Ganglia input structure, is important for the learning and execution of movement sequences. Striatal medium spiny neurons (MSNs) form cell assemblies whose population firing rates vary coherently on slow behaviourally relevant timescales. It has been shown that such activity emerges in a model of a local MSN network but only at realistic connectivities of 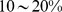 and only when MSN generated inhibitory post-synaptic potentials (IPSPs) are realistically sized. Here we suggest a reason for this. We investigate how MSN network generated population activity interacts with temporally varying cortical driving activity, as would occur in a behavioural task. We find that at unrealistically high connectivity a stable winners-take-all type regime is found where network activity separates into fixed stimulus dependent regularly firing and quiescent components. In this regime only a small number of population firing rate components interact with cortical stimulus variations. Around 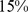 connectivity a transition to a more dynamically active regime occurs where all cells constantly switch between activity and quiescence. In this low connectivity regime, MSN population components wander randomly and here too are independent of variations in cortical driving. Only in the transition regime do weak changes in cortical driving interact with many population components so that sequential cell assemblies are reproducibly activated for many hundreds of milliseconds after stimulus onset and peri-stimulus time histograms display strong stimulus and temporal specificity. We show that, remarkably, this activity is maximized at striatally realistic connectivities and IPSP sizes. Thus, we suggest the local MSN network has optimal characteristics – it is neither too stable to respond in a dynamically complex temporally extended way to cortical variations, nor is it too unstable to respond in a consistent repeatable way. Rather, it is optimized to generate stimulus dependent activity patterns for long periods after variations in cortical excitation.

## Author Summary

The striatum forms the main input to the Basal Ganglia (BG), a subcortical structure involved in reinforcement learning and action selection. It is  composed of medium spiny neurons (MSNs) which inhibit each other through a network of collaterals, receive excitatory projections from the cerebral cortex, and are the only cells which project outside the striatum. Because of its inhibitory structure, the MSN network is often thought to act selectively, transmitting the most active cortical inputs downstream in the BG while suppressing others. However, studies show that local MSN network connections are too sparse and weak to perform global selection and their function remains puzzling. Here we investigate a different hypothesis. Rather than generating a static stimulus dependent activity pattern, we suggest the MSN network is optimized to generate stimulus dependent dynamical activity patterns for long time periods after variations in cortical excitation. We demonstrate, using simulations, that the MSN network has special characteristics. It is neither too stable to respond in a dynamically complex temporally extended way to cortical variations, nor is it too unstable to respond in a consistent repeatable way. We discuss how these properties may be utilized in temporally delayed reinforcement learning tasks strongly recruiting the striatum.

## Introduction

The striatum forms the main input to the Basal Ganglia (BG), a subcortical structure involved in reinforcement learning and action selection. It is 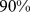 composed of medium spiny neurons (MSNs) which inhibit each other through a local network of collaterals, receive excitatory projections from the cerebral cortex and are the only cells which project outside the striatum. Because of its inhibitory structure the MSN network is often thought to act selectively, transmitting the most active cortical inputs downstream in the BG while suppressing others. However studies show that local MSN network connections are too sparse and weak to perform global selection and their function remains puzzling.

Many studies of neural response to sensory stimuli and behavioural task events throughout the brain have found that cells display large highly repeatable variations in firing rate on slow behaviourally relevant time scales. In the striatum tonic and phasic MSN activity patterns have been observed locked to task [1]–[3] and reward predicting events [4]–[7]. Several studies show that individual MSNs display diverse response profiles with phasic activity peaks not simply at stimulus onset and offset but broadly distributed across the whole spectrum of delays after task events [8]–[10].

Since MSN network connectivity is sparse and weak it has been assumed in-vivo MSN firing patterns simply reflect cortical driving. Indeed if the roughly 10000 cortical inputs an MSN receives covary, even weakly [11], [12], on slow timescales cumulatively they could generate large modulations in MSN activity on similar time scales. It is important to understand how temporally varying cortical inputs are transformed by the MSN network and possibly interface with intrinsically MSN network generated population and cell assembly dynamics.

Indeed recent work seems to support the hypothesis that phasic in-vivo MSN activity can be partially generated internally within the striatum. Adler et al. [13] have shown that distinct coherent MSN cell assemblies are sequentially activated after sensory events. At least three different MSN clusters showed peak activity at different latencies after cue presentation in a behavioural task. The cell clusters were not differentiated by intrinsic cell properties and the authors suggested their dynamics might be MSN network generated. Indeed the BG have a strongly convergent largely feed-forward architecture. Although MSNs may be unable to inhibit downstream targets in the GPe and SNr individually they may be able to do so by acting coherently in such cell assemblies.

Several other recent studies have suggested the possibility that rather than acting independently MSNs may act coherently in cell assemblies [14], [15]. Cell assembly activity is commonly observed throughout the brain [14]–[22]. However in contrast to cortical studies where cell assemblies are often defined through precise repetitive spiking relationships striatal studies suggest that MSNs do not synchronize on precise timescales but rather display coherently varying firing rates generated by coherent burst firing episodes on slower timescales [14], [15], [23]. In-vitro investigations [14], [24], [25] found that MSN cell assemblies fire coherently in recurrent sequential episodes and generate complex spatio-temporal patterns while network transition matrices display abrupt transitions between different active cell assemblies.

In recent modeling work [26] we showed that the local MSN network even when driven by constant cortical excitation can generate such slowly varying cell assembly dynamics providing cells are excited just above firing threshold. In this ‘balanced’ situation even small changes in network generated inhibition or cortical excitation can cause cells to switch between firing and quiescent states. Network generated activity was in close agreement with experiment only at striatally relevant connectivity of around 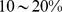.

Here we investigate how sudden switches in cortical driving, as might occur in sensory driven behavioural tasks interacts with MSN network generated chaotic cell assembly activity. We show that stimulus specific cell assemblies can be reliably activated in sequence locked to stimulus switch times, resulting in slowly varying peri-stimulus time histograms (PSTH). Thus rather than generating a static stimulus dependent activity pattern we suggest the local MSN network is optimized to generate stimulus dependent dynamical activity patterns for long time periods after variations in cortical excitation. We investigate how this activity depends on network parameters and find that MSN task modulation is optimized in a marginally stable transition regime which occurs at striatally relevant connectivities and synaptic strengths. We discuss how these properties may be utilized in temporally delayed reinforcement learning tasks strongly recruiting the striatum.

## Results

### Networks display stimulus switching induced reproducible patterns

In this section we illustrate stimulus onset locked cell assembly dynamics using an example time series. We show that the MSN network can generate prolonged sequences in response to sudden changes in otherwise constant cortical stimuli. Thus we show that the MSN network produces a dynamic sequence rather than a static state of active and quiescent cells due to the MSN network dynamics rather than the cortical drive.

**Figure 1:**
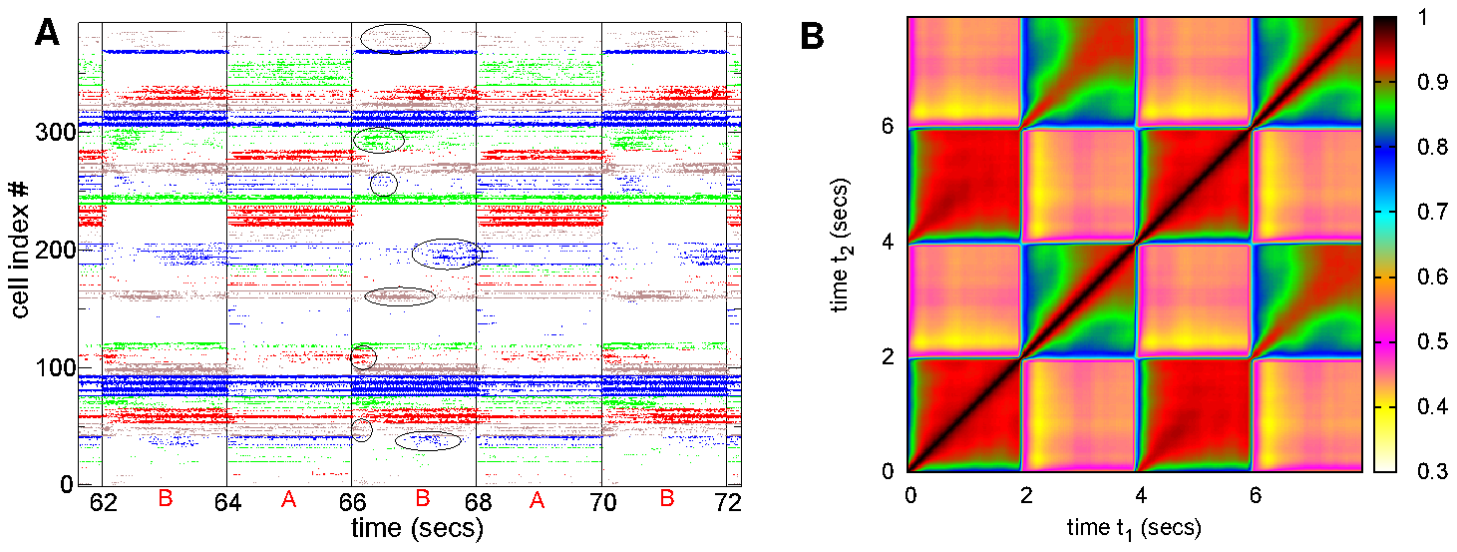
Stimulus onset locked reproducible cell assembly sequences. Cell raster plot time series segment for the 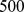 cell network simulation with connectivity 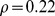, inhibitory neurotransmitter timescale timescale 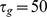 msec and synaptic strength parameter 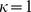 so that peak synaptic conductance is 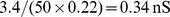 and peak IPSP size 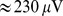, corresponding to Figure 8(e,f). 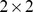 second input switching stimuli 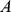 and 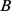 are indicated on bottom axis. Cells are grouped and coloured by k-means clusters with 30 clusters applied to only stimulus 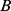. All cells active in stimulus 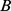 shown. Elipses indicate cell cluster bursts which appear to repeat across multiple presentations of stimulus 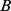. (b) 8 second similarity matrix 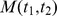 averaged across the whole 180–12 second time series, including 42 presentations of each stimulus, a segment of which is shown in (a). Colours shown in key. Stimulus A is presented during periods 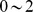 and 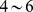 and stimulus B is presented during periods 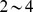 and 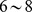 secs.

In Figure 1(a) we show a spike raster plot from a 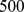 cell MSN network simulation of connectivity 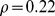. The simulation is subject to an input switching protocol where two different stimuli, each characterised by a fixed set of cortical input rates (see Methods), are applied for two seconds each in alternation repeatedly. Cells have been ordered by a clustering algorithm (see Methods) applied to only one of the stimuli, B, and each of the 30 clusters is coloured differently. As can be seen individual cells fire spikes in episodic bursts lasting up to many hundreds of msecs. The MSNs fire approximately periodically with period two seconds, the period of the forcing stimulus. Most cells do not fire throughout the whole duration of a stimulus but ‘phasically’ at specific epochs often several hundred msecs after onset of a particular stimulus and lasting for only a short time.

In order to quantify the reproducibility of the dynamics we calculate the two-time firing rate *similarity* [14], [21], [22], [27], [28]. Similarity is just the scalar product of the vectors of cell firing rates at two different times, 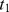 and 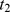. Similarity can take values ranging from 0, meaning firing rate vectors are orthogonal, to 1, meaning firing rate vectors are identical. Figure 1(b) shows a 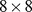 second *mean* similarity matrix, 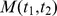 constructed by moving an eight second segment through the time series in steps of four seconds to create an average similarity with periodicity of the stimulation period (see Methods).

We denote by 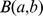 the ‘block’ of time points such that 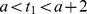 and 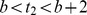. Therefore the blocks 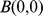, and 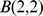 describe the mean similarity within a given presentation of respectively stimulus A or B. Sometimes this seems ‘diagonal’ (e.g. stimulus B, 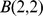) and sometimes more ‘block-like’ (e.g. stimulus A, 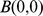). In stimulus B the similarity drops off rapidly as 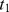 increases away from the diagonal 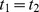 (for any 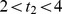) showing that the firing activity moves through a rapid succession of different states during stimulus B (as can also be observed directly in the time series Figure 1(a)). The network therefore not only represents the active stimulus but also the time elapsed since stimulus onset. On the other hand activity during stimulus A is more ‘fixed point’ like, where time elapsed from onset is not strongly encoded.

The blocks 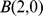 and 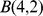 describe similarity in firing activity between a given stimulus, respectively A and B, and the immediately following stimulus, respectively B and A. As can be seen similarity is weak in these blocks demonstrating that the network activity is able to discriminate the stimuli.

The blocks 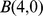 and 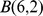 describe similarity between a given stimulus, respectively A and B and the *next* presentation of the *same* stimulus. In particular in stimulus B activity drops off rapidly as 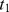 increases away from the diagonal 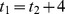 (for any 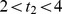) demonstrating that the network activity not only moves through a sequence of different states, but that these state sequences are *reproducible* across different presentations of a given stimulus.

These results demonstrate that an inhibitory spiking MSN network model can generate sequential patterns of activity for several hundred msecs after stimulus onset which are reproducible across different presentations of the same stimulus, but different for different stimuli. This is true even though the excitatory input strengths are fixed for the duration of a stimulus (except for random fluctuations). Thus the activation of cells is not simply determined by the input strengths. If this were the case (roughly speaking) the most strongly excited cells in any particular stimulus would remain active throughout that stimulus period while the least strongly activated would remain quiescent throughout the stimulus. Since the mean excitatory input strength is the same in both stimuli the onset locked patterns result only from the redistribution of excitation across MSNs; an increase in mean excitation level is not required. This is because cells are balanced close to firing threshold where even small variations in input drive cause a large change in the distribution and temporal evolution of activity across the inhibitory asymmetrically connected network. Thus balanced network activity provides cells with a large diversity of strong temporal responses to a given stimulus, rather than generating a static state of active and quiescent cells. Moreover clusters formed from many cells can also display this behaviour as observed in the time series Fig. 1(a).

Recognition of stimuli through sequential activations remains stochastic however; on some trials a stimulus fails to generate its normal patterns. These failures may correspond to error trials in a behavioural task. Stochastic stimulus recognition is not due to the random fluctuations in excitation, but an effect of the chaotic network dynamics, also occurring in deterministic spiking network simulations as described in Supplemental Text S1. These results extend those briefly reported in our previous publication [29]. In the following we investigate why this activity occurs and under what MSN network conditions it occurs maximally.

### Stimulus onset locked reproducible dynamics optimized near striatal connectivity

We have demonstrated that stimulus onset locked reproducible dynamics can occur in network simulations, but how does it depend on the network parameters such as connectivity and connection strength? To investigate these issues quantitatively we calculate mean similarity profiles for simulations of 500 cell networks. In previous work [26] (and see Model) we have suggested that a 500 cell network can provide a reasonable representation of real MSN network activity. This is because it respects both the striatally relevant MSN connection probability, of about 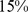, and the approximate number of cells, 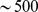, contacted by a given MSN since only a proportion of the MSN cells 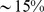 are depolarized to firing threshold by cortical excitation. We demonstrate here that the reproducibility of stimulus onset locked dynamics is maximized at striatally relevant connectivities.

**Figure 2:**
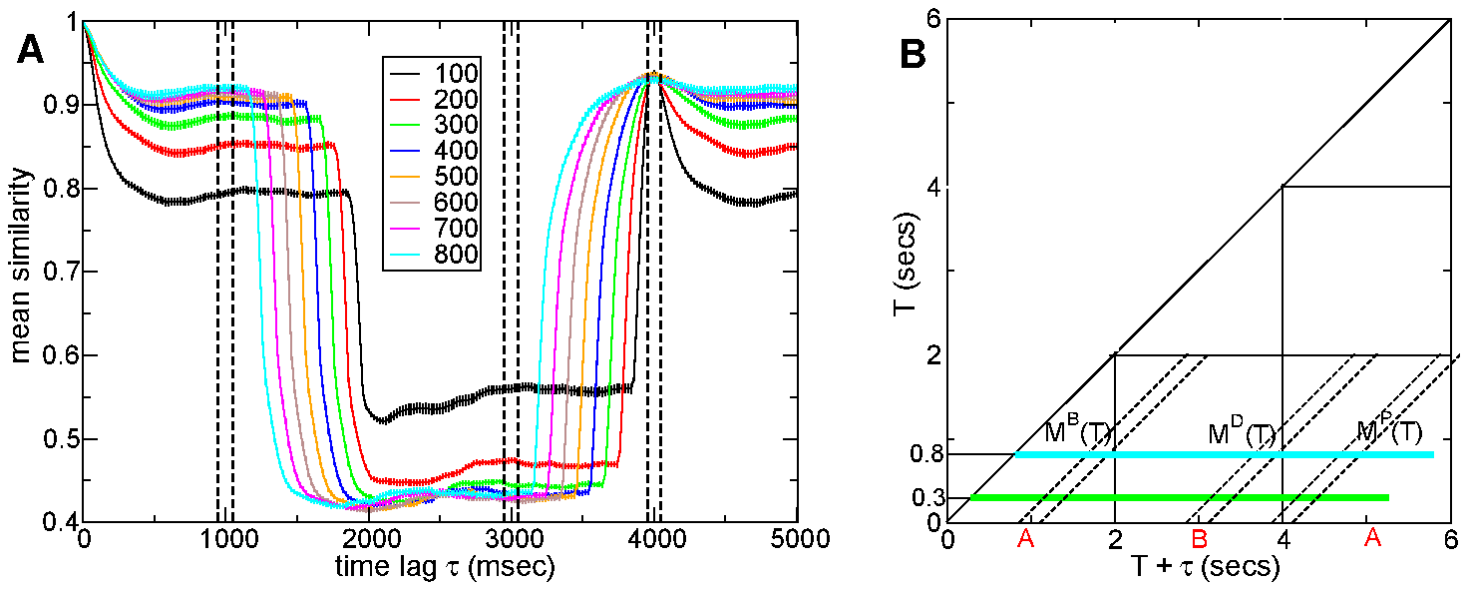
Mean firing rate similarity shows peak at same time epoch in the following presentation of current stimulus. (a) Mean similarity profiles 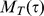 for connectivity 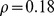 network simulation versus time lag 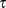. Firing rate similarities calculated using 100 msec window incremented in 10 msec steps. Epochs 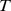 after stimulus onset shown in key. Bars indicate sem. Vertical lines indicate averaging periods (see Figure 2(b)). 500 cell network simulation of length 180–12 seconds under a 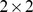 second input switching protocol. Inhibitory neurotransmitter timescale 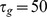 msec. Synaptic strength 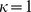 so that peak synaptic conductance is 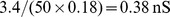 and peak IPSP size 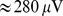 (b) Illustration of mean similarity profiles 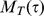 and calculation of averages 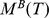, 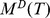, 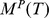. For example the green solid line shows 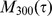 while 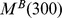 is the mean similarity in the intersection of the green solid line and the two diagonal lines denoted by 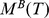 at time lags 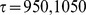.

Figure 2(a) shows cross-sections from mean similarity matrices 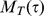 (see Methods and Figure 2(b)) calculated from a 500 cell connectivity 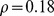 network simulation of 180 seconds, after discarding a 12 second transient. As in the example time series above (Figure 1(a)) here network simulations have two stimuli, each presented for two seconds, alternately. Each profile shows the similarity between a 100 msec window centered on a given epoch 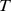 msecs after the onset of a stimulus and another 100 msec window at a later time 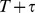, averaged across all presentations of both stimuli. The time lags 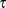 extend for 5 seconds, that is to a point near the end of the next presentation of the current stimulus. In other words these are profiles along a horizontal (or vertical) slice from the point 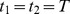 through a mean similarity matrix like the one shown in Figure 1(b) as schematically illustrated in Figure 2(b).

The late epoch 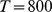 msec similarity profile, Figure 2(a, cyan), describes how similarity behaves far from stimulus onset. After about half a second (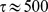 msec) firing activity patterns decorrelate and similarity decays to its background level of about 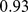. This is the level of similarity between firing activity patterns separated by long time periods under a constant stimulus. At time lag 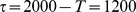, the switch to a different stimulus occurs. As can be seen this 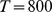 similarity profile (cyan) shows a sudden change to a lower level around 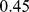. This low level of similarity, in this case close to the similarity level 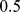 of uncorrelated activity, demonstrates that the different stimuli evoke very different activity patterns. At time lag 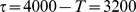 the onset of the next presentation of the same stimulus occurs and similarity returns to its background level of around 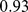. Similarity shows a (broad and weak) peak centered exactly on time lag 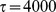 msec. Thus activity is most similar at the same epoch 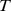 in the next presentation of the current stimulus, even at this late epoch 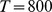 msec after stimulus onset. The existence of this peak demonstrates that the dynamical evolution after stimulus onset is reproducible across presentations.

The behaviour is different at epochs 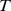 close to stimulus onset, such as 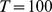 msec (black). Activity in this early epoch is much less similar to the stimulus' background activity, as shown by the decay to a much lower level of similarity (around 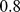) than the 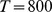 epoch (cyan) case. At time lag 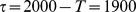, the switch to a different stimulus occurs. Similarity drops to a lower level, but not as low as the epoch 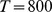 (cyan) level. Thus firing activity early after a stimulus switch is more similar to the previous (and subsequent) stimulus than later after the switch (see Discussion). Again similarity shows a peak at 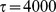, the exact same epoch 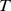 in the next presentation of the current stimulus. This 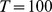 epoch similarity peak is much sharper than the 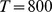 (cyan) one. Similarity profiles 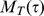 at intermediate 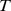 show decreasing 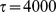 peak sharpness with increasing 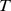 indicating that the reproducibility of the dynamical evolution does not continue indefinitely.

We now investigate how reproducibility of dynamical evolution depends on network connectivity. As explained in the Model section when we vary connectivity 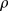 we rescale the connection strength by the connection probability so that the mean level of inhibition on a cell is unchanged by the connectivity variation.

The reproducibility at epoch 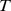 of the stimulus onset locked dynamics can be quantified by the difference in the height of the 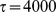 peak seen in Figure 2(a) and the background level as a function of epoch 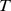. Indeed if the stimulus onset locked dynamical evolution were not reproducible at a given epoch 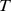 then the epoch 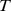 similarity profile would not show a 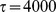 peak and similarity would remain at the peak level of 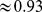, like the 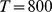 (cyan) similarity profile does. Thus we calculate the average mean *background* similarity, 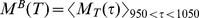 and *peak* similarity 
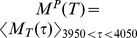
 obtained by averaging 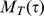 over the time lag 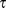 ranges 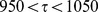 and 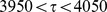, respectively, (shown by the vertical lines in Figure 2(a) and illustrated schematically in Figure 2(b)) for different epochs 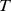 after stimulus onset.

**Figure 3:**
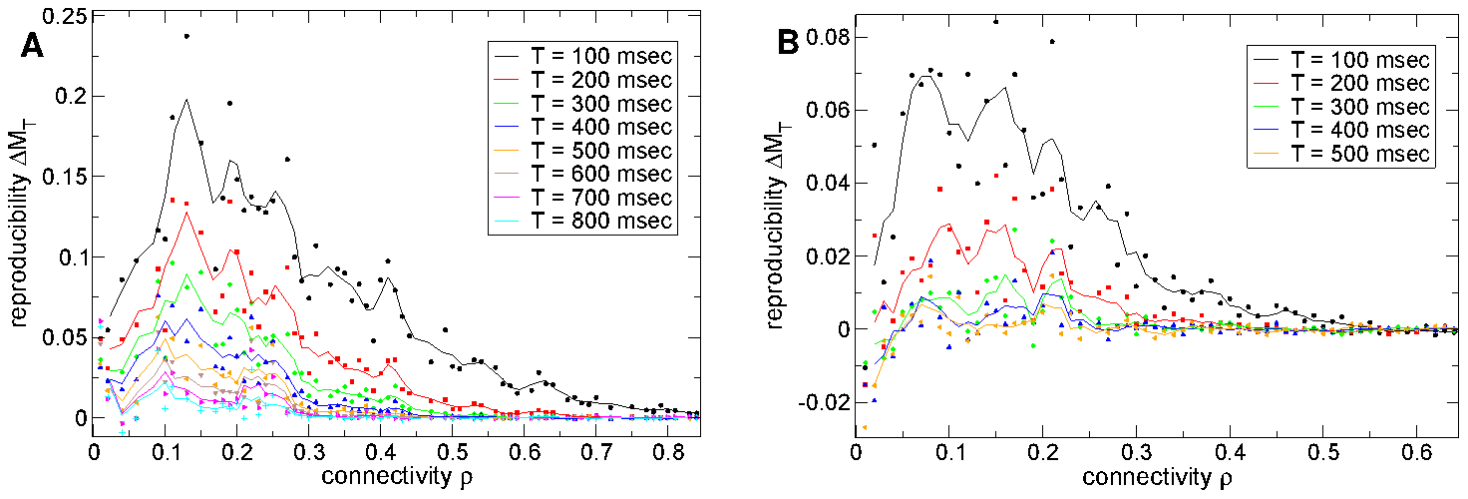
Stimulus onset locked reproducible dynamics maximal at striatal connectivity. (a) Strength of stimulus onset locked reproducible dynamics 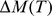 (see text) versus connectivity 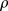 for several different epochs 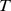 after stimulus onset (see key) corresponding to Figure 2(b). Inhibitory neurotransmitter timescale 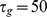 msec. Synaptic strength parameter 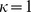 so that peak synaptic conductance varies as 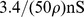 and peak IPSP size as 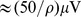. (b) Same as (a) except inhibitory neurotransmitter timescale reduced by 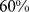 to 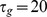 msec. Synaptic strength parameter 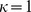 so that peak synaptic conductance varies as 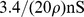 and peak IPSP size as 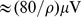. (a,b) 500 cell network simulations of length 180–12 seconds under the 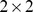 second input switching protocol. Points show actual values, solid lines show three point average.

The quantity 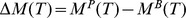, is plotted versus connectivity for several epochs 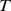 in Figure 3(a). At high connectivity 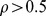, 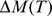 approaches zero for all epochs 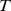. Below this connectivity it starts to increase, displaying a peak around connectivity 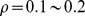 before decreasing again. Around connectivity 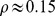, 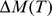 is significantly greater than zero up to about epoch 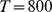 msec (cyan line) indicating reproducible stimulus locked dynamics persists for this long after stimulus onset at this connectivity. Most interestingly reproducible stimulus locked activity appears optimal at connectivities close to real striatal connectivity.

### Peak in dynamical reproducibility is robust to decrease in time scale of inhibition

In the Model section we explain that the time scale of inhibitory neurotransmitter decay is set by the parameter 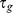. In the above this has been set to 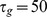 msecs in accordance with Janssen et al. [30] which shows a time course of MSN IPSP with a half life of recovery of about 30–40 msec. However a fairly large range of values has been found in various studies depending on experimental conditions [31]–[35]. Here we investigate network behaviour when 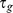 is reduced to 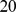 so that the decay half-life 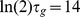 msec.

Figure 3(b) shows the same computation of the reproducibility of stimulus onset locked dynamics 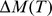 shown in Figure 3(a) except using the reduced setting for 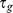. Evidently 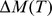 shows a very similar behaviour at this lower 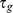, including the peak around connectivity 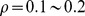. The magnitude of the effect is much reduced however as can be seen by the peak height. Furthermore even at optimal connectivity, 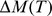 is only significantly different from zero up to about epoch 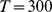 msec. However the results presented above, in particular the peak in 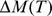 at striatally relevant connectivity are robust to at least 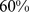 reduction in 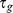.

### Peaks in dynamical reproducibility and distinguishablity occur when inhibitory connections have near striatal strength

**Figure 4:**
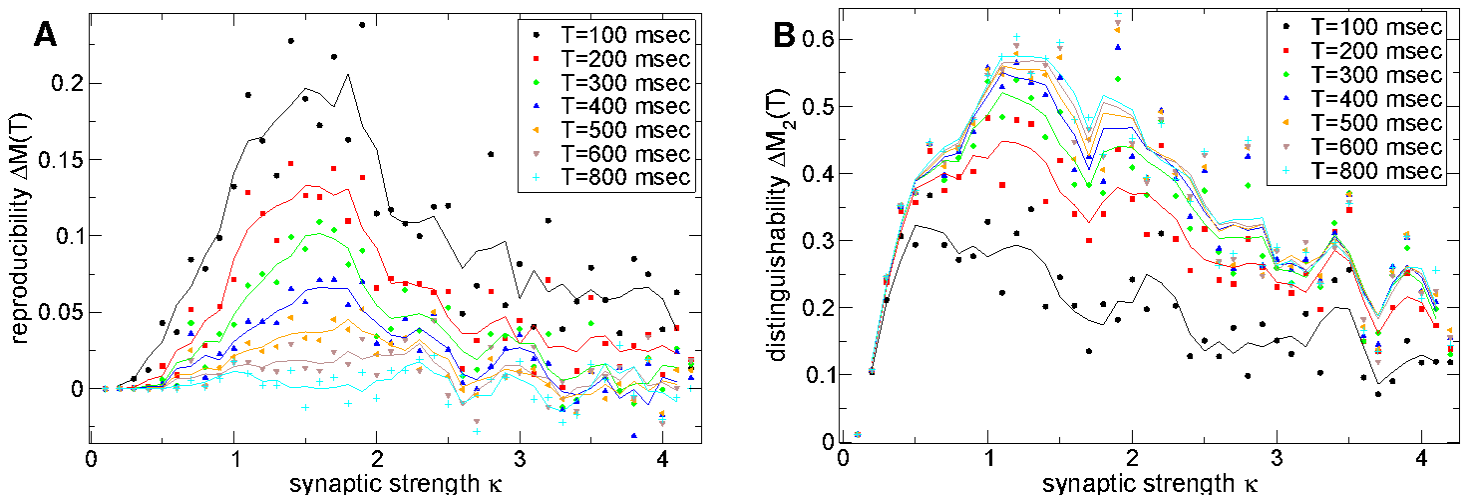
Stimulus onset locked reproducible dynamics and stimulus distinguishability maximal at striatal connection strengths. (a) Strength of stimulus onset locked reproducible dynamics 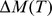 (see text) versus synaptic strength parameter 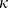 for connectivity 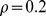 and timescale of inhibitory neurotransmitter 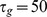 msec. Actual peak conductance is given by 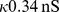 and 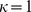 generates realistic peak IPSP sizes of around 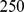 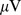. Several different epochs 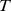 after stimulus onset (see key) for 500 cell network simulations of length 180–12 seconds under a 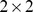 second input switching protocol. Points show actual values, solid lines show three point average. (b) Same as (a) but stimulus distinguishability 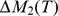.

We can also ask how the reproducibility of stimulus onset locked dynamics, 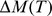, depends on the strength of inhibitory connections. In the Model section we explain that the connection strength parameter 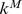 was chosen to be 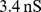 in order to generate realistic IPSPs of around 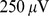 [26] at connectivities around 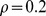 when the postsynaptic cell is close to firing threshold and the inhibitory neurotransmitter timescale has the value 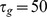 msec. At these parameter values the peak conductance generated by a spike is 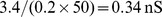 (see Model section.) Here we fix the connectivity 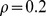 and timescale 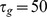 and vary the synaptic strength around the value which produces IPSPs of realistic size. Thus the peak conductance is set to be 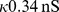 and 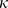 varied so that 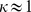 recovers IPSPs of realistic size. In Figure 4 we show that variation with 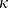 also produces a peak in 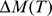 for epochs up to about 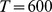 msec after stimulus onset. The peak is very close to 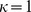. Remarkably the maximum occurs close to the value of connection strength which recovers IPSPs close to experimentally observed size.

In Figure 4(b) we also show a stimulus *distinguishability* measure 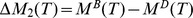. Here the *different* stimulus similarity, 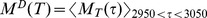 is obtained by averaging 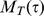 over the time lag 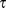 range, 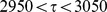 (shown by the vertical lines in Figure 2(a) and illustrated in Figure 2(b)). The distinguishability of background activities under the two stimuli is given by the large epoch 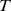 results, for example by 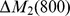. A value of zero indicates that similarity between firing activity at two well separated time points in a given stimulus is the same as between two different stimuli, and thus activity is solely dependent on the network irrespective of the stimulus. Stimulus distinguishability 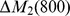 (cyan) also remarkably has a peak near 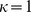 in the striatally relevant region. For shorter epochs after stimulus onset, for example 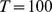 (black), the quantity 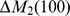 is smaller because soon after stimulus onset firing activity resembles the previous stimulus. Stimuli become more distinguishable as time elapses (see Discussion.)

### MSN network shows dynamical regime transition as connectivity and connection strength are varied

We have shown that the reproducibility of stimulus onset locked dynamical evolution and stimulus distinguishability are optimized in the striatally relevant parameter region of connectivity and connection strength. We now investigate why this should be. Here we show that the peaks occur near a transition in network activity which occurs in the striatally relevant parameter region and demonstrate the nature of the transition. In this section we investigate 500 cell network simulations under *constant* (randomly fluctuating) excitatory drive *without* the stimulus switching.

**Figure 5:**
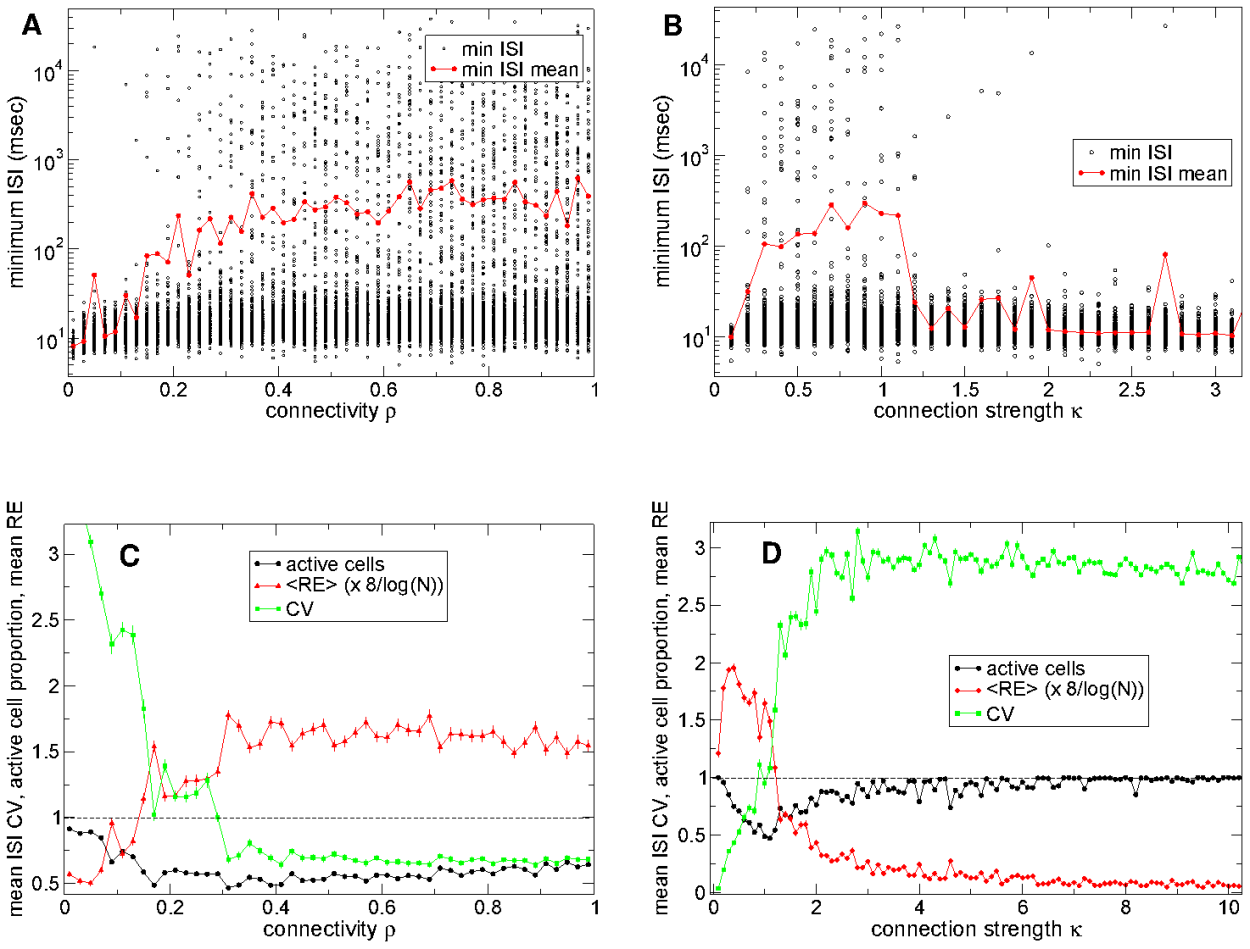
Dynamical regime transition in network activity. (a) Black circles: minimum observed ISI for each active cell in network simulations of different connectivity. Red line: mean of minimum observed ISI across all cells for each network simulation. Synaptic strength parameter 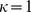 so that peak synaptic conductance varies as 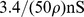 and peak IPSP size 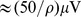. (b) Same as (a) but versus synaptic strength parameter 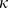 for connectivity 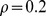. Actual peak synaptic conductance is given by 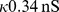 and 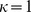 generates realistic peak IPSP sizes of around 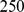 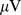. (c) Green line: mean ISI coefficient of variation (CV) across all cells in network simulations of different connectivity corresponding to (a) (bars indicated sem). Black line: proportion of active cells (those that fire at least three spikes in the 168 second time series). Red line: mean relative entropy, 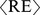 of 100 msec firing rate distribution across all cells rescaled by 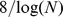 where 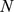 is the number of active cells (see text) (bars indicated sem). (d) Same as (c) but corresponding to (b). (a,b,c,d) 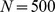 cell network simulations under constant (randomly fluctuating) excitation without stimulus switching. 180–12 second time series. Inhibitory neurotransmitter timescale 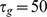 msec.

The black points in Figure 5(a) show the minimum inter-spike-interval (ISI) observed for each active cell (cells which fire at least three spikes in the 168 second observation period) in network simulations of different connectivity. At high connectivity the distribution is very broad. Most cells have minimum ISIs of between 10 and 20 msecs but many have much longer minimum ISIs. This indicates that at high connectivity the network displays winner(s)-take-all like activity.

On the other hand at low connectivity the minimum ISI distribution does not show the quiescent component. The transition from a broad distribution to a narrow one appears to occur fairly suddenly around 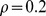 connectivity. This is also observed in the mean minimum ISI (Figure 5(a) red line) which is roughly flat with high value above 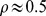 connectivity, but falls off rapidly below around connectivity 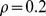.

The coefficient of variation (CV) of a cell's ISI distribution, defined as the cell's ISI standard deviation normalized by its mean ISI, also reveals the connectivity dependent transition. Figure 5(c, green line) shows how this quantity, averaged across all active cells, varies with network connectivity corresponding to Figure 5(a). At connectivities above around 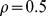 it is roughly flat with value around 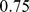 indicating that on average cells are firing fairly regularly. Below about connectivity 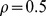 it starts to increase and very rapidly below about connectivity 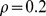. Spike time series' become significantly more bursty than Poissonian (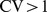) around 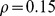 connectivity. Thus we find a transition from a network state where the active cells fire mostly regularly to a state where active cells fire in an episodic bursting way.

The proportion of active cells (those that fire at least three spikes in the 168 second observation period) also demonstrates the connectivity dependent transition. This quantity (Figure 5(c), black line) shows a minimum around connectivity 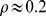 where about 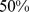 of the network cells are active. On increasing connectivity the active proportion rises slowly towards about 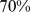 at full connectivity while on decreasing connectivity it rises rapidly towards 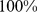 activity at zero connectivity. Indeed when fewer cells are active we expect network generated fluctuations to be reduced and the remaining active cells thus fire more regularly, reducing the CV values at higher connectivity.

Thus the network shows a fairly sharp transition from a regularly firing winners-take-all type regime where a proportion of cells are permanently quiescent to a regime where almost all cells are involved in bursty activity. Remarkably actual striatal connectivity of around 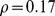 appears to be in the transition regime.

Figure 5(b,d) show the same quantities but versus the connection strength parameter 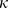 for network simulations of connectivity 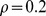. Again network dynamics shows a transition. In the approximate region 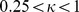, (so that peak IPSP sizes vary between 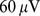 and 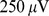 and peak synaptic conductances vary between 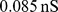 and 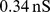), the network shows a winners-take-all behaviour. This can be seen from the broad distribution of minimum ISI (Figure 5(b), black points) with some very long minimum ISI, the very high mean ISI (Figure 5(b), red line), the proportion of active of cells (Figure 5(d), black line) indicating that less than 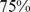 of the network is active, and the low mean ISI 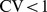 (Figure 5(d), green line), indicating that network simulations include many relatively regularly firing cells. At higher 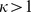, (peak IPSP size 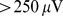 and peak synaptic conductance 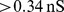) on the other hand, the network appears to be in a highly active state with many burst firing cells. This is indicated by the high ISI 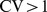, the narrow distribution of minimum ISI with low mean ISI and the fact that most of the network cells are active.

At very low 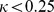 (peak IPSP size 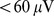 and peak synaptic conductance 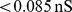) however we find another regime where connection strength vanishes and thus all cells in the network fire perfectly regularly (except for stochastic fluctuations in excitatory input).

Remarkably again the transition between the winners-take-all like regime and the bursty active regime appears to be close to 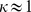, in the striatally relevant parameter region where presynaptic spikes generate realistically sized IPSP 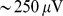. Notice also that in both the connectivity 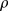 variation and synaptic strength 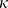 variation the transition occurs close to a minimum of approximately 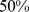 in the quantity of active cells (Figure 5(c,d) black lines). This is also where the mean ISI CV is close to or slightly larger than unity (see Discussion).

**Figure 6:**
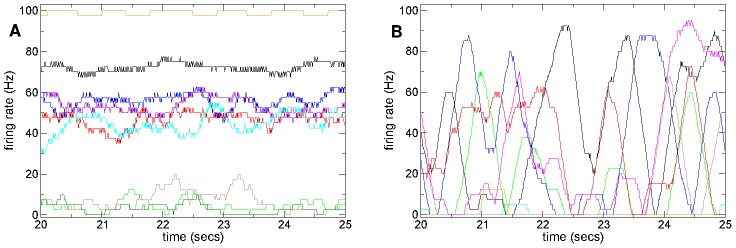
Firing rate time series show qualitatively different behaviours dependent on connectivity. (a,b) Firing rate time series segments based on 400 msec moving window for several randomly chosen cells from 500 cell network simulations under constant (randomly fluctuating) excitation without stimulus switching. Inhibitory neurotransmitter timescale 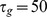 msec. (a) Connectivity 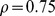, synaptic strength parameter 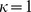 so that peak synaptic conductance is 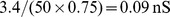 and peak IPSP size 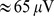; (b) Connectivity 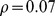, synaptic strength parameter 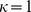 so that peak synaptic conductance is 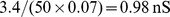 and peak IPSP size 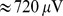.

Finally, as an illustration of the different activity in the two regimes, Figure 6 shows rate time series for several cells from a high connectivity (Figure 6(a)) simulation in the winners-take-all regime and a low connectivity (Figure 6(b)) simulation in the active bursty regime. As can be seen, in the winners-take-all regime (Figure 6(a)) firing rates seem to fluctuate mildly around well-defined seemingly stable mean levels. Individual cells appear to have narrow firing rate distributions which overlap only weakly with other cells rate distributions. In contrast in the bursty regime firing rates fluctuate wildly between zero and maxima defined by the cells driving cortical excitations, and appear very unstable, so that cells have broad strongly overlapping rate distributions.

This observation can be quantified by the relative entropy 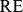 (see Methods) between a cell's firing rate distribution and the *combined* firing rate distribution of all active cells in a given network simulation. This relative entropy is zero when the firing rate distribution of a single cell coincides with the combined firing rate distribution across all cells. On the other hand it reaches a value 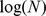 when the firing rate distributions of the 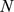 active cells are entirely non-overlapping. The quantity 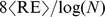 (the factor 8 is included simply for convenient scaling on the figure) is shown averaged across all cells in the network versus connectivity in Figure 5(c) and versus connection strength in Figure 5(d) by the red lines. As can be seen it also exhibits the transition at striatal relevant parameter settings of 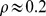 and 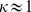. At lower connectivity or higher connection strength the firing rate distributions of single cells are similar to the distribution across all cells combined. In contrast at higher connectivity or lower connection strength the rate distributions of individual cells are much less overlapping.

### Rate dynamics is marginally stable at striatally relevant connectivity and connection strength

Above we have shown that the MSN network displays a transition between a bursty active regime and a winners-take-all like regime as connectivity and connection strength are varied. The transition occurs at striatally relevant parameter settings. Here we demonstrate that the rate dynamics generated by the MSN network model is unstable and chaotic in the bursty active regime but stable in the winners-take-all like regime and thus marginally stable at striatally relevant parameter settings.

The postsynaptically bound inhibitory neurotransmitters 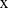 vary slowly in the MSN network model [26] and essentially act as a low-pass filter of presynaptic spiking activity [26]. By replacing the detailed dependence on presynaptic activity with the presynaptic firing rate we obtain a reduced rate model describing the dynamical activity of the postsynaptically bound inhibitory neurotransmitters 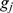 (see Methods.) The reduced model has exactly the same parameters as the full spiking network model including the inhibitory connectivity structure and excitatory driving. However in order to study the stability of network generated deterministic rate dynamics the noise in the excitatory driving is not included. Again the excitatory driving is fixed for the duration of the simulation without stimulus switching. The conductance based synapses are also replaced by current synapses which do not depend on the postsynaptic membrane potential.

**Figure 7:**
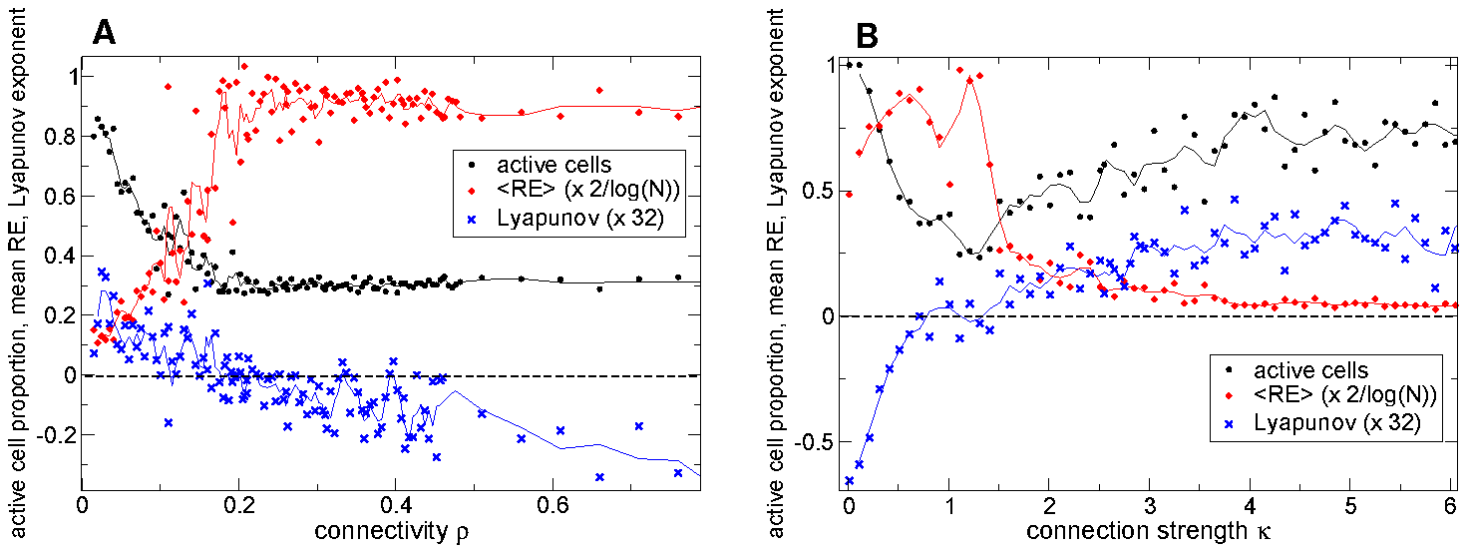
MSN network rate dynamics is marginally stable at striatally relevant connectivity and connection strength. (a,b) Black circles : proportion of active cells. Red diamonds : mean relative entropy 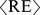 rescaled by 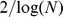 where 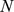 is the number of active cells. Blue crosses : maximal Lyapunov exponent rescaled by 32. Solid lines show three point averages. (a) Variation in connectivity 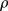 for many simulations. Synaptic strength parameter 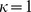 so that peak synaptic conductance varies as 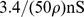 and peak IPSP size as 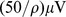 (b) Variation in connection strength 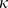 for many simulations of connectivity 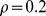. Actual peak conductance is given by 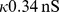 and 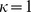 generates realistic peak IPSP sizes of around 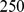 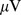. (a,b) 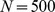 cell deterministic reduced rate network simulations (see main text) of length 110 secs. Initial 100 secs discarded from analysis. Inhibitory neurotransmitter timescale 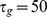 msec.

The deterministic rate model shows a very similar qualitative dependence of the number of active cells on connectivity (Figure 7(a) black circles) and connection strength (Figure 7(b) black circles) as the full spiking model (Figure 5(c,d)). A weak minimum is shown at striatally relevant connectivity around 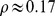 and a marked minimum at striatally relevant connection strength 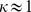. The same is true for the variation of the relative entropy 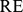 with connectivity and connection strength, (Figure 7(a,b) red diamonds.) As in the full spiking model a fairly sudden transition is seen at striatally relevant connectivity and connection strength.

The reduced model is deterministic and since it also lacks the strong instability of the spike generating mechanism we are able to compute the maximal Lyapunov exponent for the rate dynamics of 500 cell networks. This quantity characterises the stability of the rate dynamics. When it is positive the network rate dynamics is chaotic. When it is negative however the network has a found a fixed distribution of firing rates or alternatively some, or all, of the cells firing rates may be varying periodically. As can be seen by the blue crosses in Figure 7(a,b) network rate dynamics is unstable and chaotic in the active bursty regime but stable in the winners-take-all regime. Only in the striatally relevant parameter regime is the maximal Lyapunov exponent close to zero indicating the network is marginally stable. This point is also known as the ‘edge-of-chaos’ [36]–[39]. The other quantities, the relative entropy 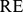 and the proportion of active cells show strong fluctuations across simulations when the Lyapunov exponent is close to zero. This is due to the simultaneous proximity of stable and unstable states.

Time series examples from simulations of this reduced rate model displaying fixed point, periodic and chaotic activity are shown in Supplemental Text S1. The distribution of fixed point, periodic and chaotic states under variation of connectivity 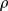 and connection strength 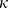 is also shown in Supplemental Text S1.

### Stimulus onset locked reproducible dynamics mediated by coherently activating cell populations

Above we have demonstrated that temporally extended reproducible sequential dynamics can occur locked to stimulus switches. We have shown this activity occurs maximally near a transition in network activity where rate dynamics is marginally stable and which occurs in the striatally relevant parameter range. However in principle sequential activity could be mediated by a chain of single cells activated in sequence. Coherent activity of cell assemblies [14], [15], [23]–[25] has also been observed in the striatum and such population activity could provide a potent force to inhibit and disinhibit downstream targets. Here we investigate whether stimulus onset locked sequential dynamics is also shown by cell assemblies, as well as by individual cells.

The cell spike raster plot time series segment from the intermediate connectivity, 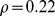, 500 cell simulation shown in Figure 1(a) seems to indicate that reproducible stimulus onset locked dynamics is indeed mediated by cell assemblies rather than single cells. Indeed the network appears to switch through different sequentially activated distributions of active and quiescent cell assemblies (indicated by ellipses) throughout stimulus 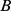, which approximately repeat across different presentations of stimulus 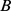. On the other hand, at high and low connectivity reproducible sequentially activated distributions of active and quiescent cells are not observed (see time series described in Supplemental Text S1.)

To investigate this further here instead of using k-means clustering we employ principal component analysis (PCA) of 500 cell network simulations. Principal components are linear combinations of single cell firing rates with fixed coefficients such that the resultant component activity time series are uncorrelated with each other. PCA is closely related [40] to the k-means clustering methodology used as an illustration of time series above (Figure 1(a)) but is non-parametric and does not require either a choice of cluster quantity nor does it depend on the initial conditions of the algorithm. Like k-means clusters components are generated from the correlation matrix of firing rates of all active cells based on a long 100 msec time window. Thus components here do not reflect precise spiking relationships. Rather principal component time series can be considered to describe population firing rates where however cells can contribute both positively or negatively to any component. When the components are ordered by variance of their rate time series', largest first, the smallest numbered (highest) components are the ones containing the major contributions to the variance.

**Figure 8:**
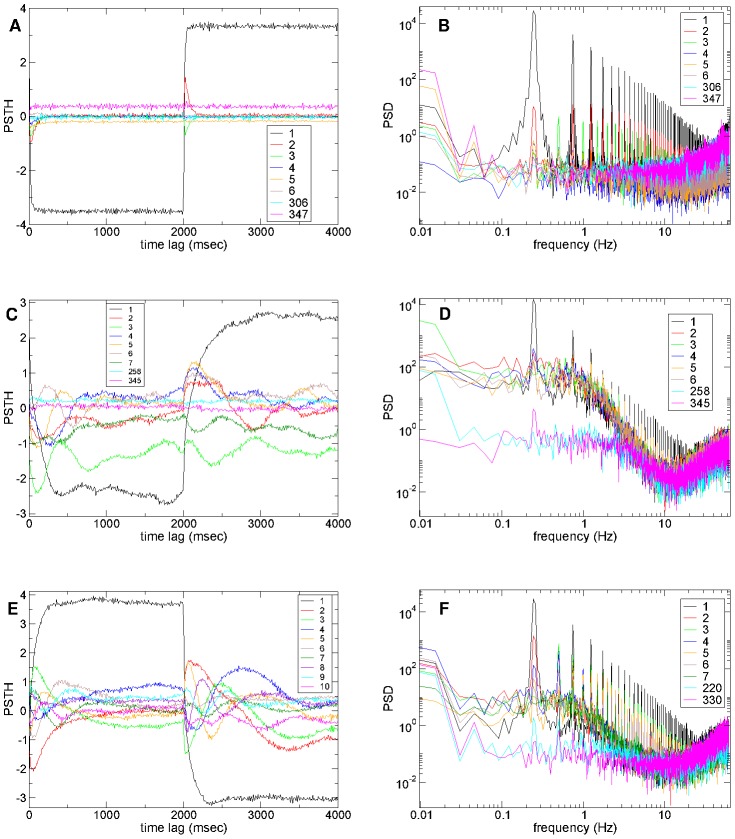
Population component dynamics shows strong stimulus interaction at intermediate connectivity. (a,c,e) PSTH for several principal components (see key) locked to stimulus onset in the 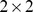 second input switching protocol calculated from 180–12 second time series including 42 presentations of each of the two stimuli. 500 cell network simulations. Synaptic strength parameter 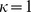. Inhibitory neurotransmitter timescale 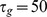 msec. (b,d,f) PSD of components corresponding to (a,c,e) in log-log axes. (a,b) High connectivity 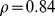, so that peak synaptic conductance is 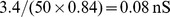 and peak IPSP size 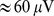; (c,d) low connectivity 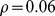, so that peak synaptic conductance is 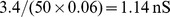 and peak IPSP size 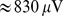; (e,f) intermediate connectivity 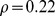, so that peak synaptic conductance is 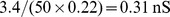 and peak IPSP size 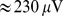.

Using component analysis we can demonstrate that network dynamics can evolve in a much smaller dimensional space than the number of cells [41], [42]. Figure 8(a,c,e) show peri-stimulus time histograms (PSTH) of component time series calculated in exactly the same way as PSTH for single cell time series for high, low and intermediate connectivity simulations under the 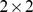 second input switching protocol used above (see Methods).

At high connectivity, 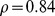, (Figure 8(a)) only the three highest components seem to show activity reflecting stimulus switching in their PSTH. The first component (black) is positively driven by cells continuously active in one stimulus and negatively driven by cells continuously active in the other stimulus. The next two components (red and green) only activate for a short period after stimulus switches. These components are composed of cells rapidly activated by the cortical stimulus but then more slowly suppressed by the winner cells composing the first component. These two components differ in that one (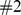, red) activates in way which is dependent on the direction of stimulus switching, while the other (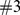, green) does not. Lower components seem to show only high frequency fluctuations and thus the dynamics is effectively only three dimensional. Thus, consistent with the transition analysis above, the dynamics at high connectivity seems to be a ‘k-winners-take-all’ state [43] where the first component (black) represents the winning set of cells. Activity seems to relax rapidly within about 50 msec after a stimulus switch suggesting that the winners-take-all state is very stable at this high connectivity. This can also be directly observed in the spike raster plot segment from this network simulation shown in Figure S1(a) of the Supplemental Text S1 and the corresponding mean similarity matrix (Figure S2(a) Supplemental Text S1).

These observations are reflected in the corresponding power spectral density (PSD) of the first ten components shown in Figure 8(b). The first two components (black and red) show a strong stimulus driven peak at 0.25 Hz while the third component (green) shows a peak at 0.5 Hz. The background activity, which is network generated, shows the flat spectrum characteristic of white noise. Much lower components, such as 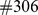 and 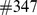 also display white noise like spectra, but with only very weak peaks.

In contrast to the high connectivity situation at very low connectivity, 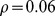, PSTH of population components (Figure 8(c)) seem to display large slow random-walk like fluctuations. The PSTH appear random even though many (here 42) stimuli presentations are averaged and the component variations appear not well-locked to stimulus onset times. These observations are also directly evident in the spike raster plot segment from this network simulation shown in Figure S1(b) of the Supplemental Text S1 and the corresponding mean similarity matrix (Figure S2(b) Supplemental Text S1).

This can also be seen by the weakening of the 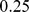 Hz, and absence of the 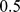 Hz peaks in the corresponding PSD of the higher components (Figure 8(d)). The network generated background activity of the higher components also shows a region of growth on intermediate frequencies 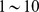 Hz, absent at high connectivity (Figure 8(b)). This will be discussed further below (see Discussion.) Thus at low connectivity, as at high connectivity, stimulus switching does not strongly interact with many components of the MSN population activity.

The situation is more interesting in the intermediate connectivity, 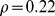, network simulation whose spike raster plot segment is shown in Figure 1(a) with corresponding similarity matrix in Figure 1(b). The PSTH of multiple components, Figure 8(e), display slow oscillations lasting over a second after stimulus onset, created by waves of inhibition and disinhibition between cell populations. The higher frequency fluctuations around these slow variations appear strongly suppressed for the first half a second after stimulus switching, compared to the low connectivity example, Figure 8(c). However after switching through several states the network does appear to eventually relax to a stable stimulus dependent equilibrium.

The increased complexity of the population dynamics is also apparent in the PSD, Figure 8(f), which shows many components with strong stimulus driven peaks at 0.25 Hz and also many with peaks at 0.5 Hz. Thus in this intermediate connectivity regime the stimulus switching interacts with many more components of the population activity than at high or low connectivity.

### Multiple population components show suppressed noise at striatal connectivity

The stimulus locking of population activity components described above can be quantified by the variance of the component PSTH fluctuations, here termed ‘PSTH variance’, and the variance of the component time series fluctuations around the mean PSTH activity, here termed ‘noise variance’ calculated across the first 400 msec after stimulus onset (see Methods).

**Figure 9:**
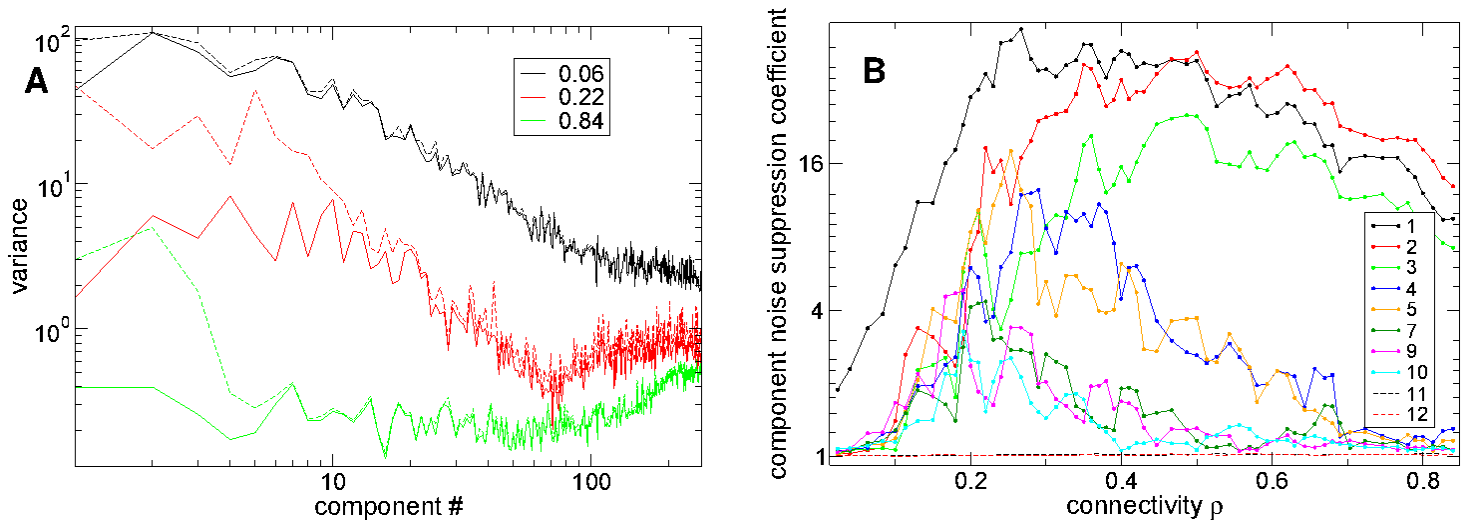
Ratio of signal to noise variance maximal at striatal connectivity in first 10 principal population components. (a) Component PSTH variance (dashed) and noise variance (solid) versus component number for three simulations of different connectivity 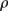 (see key) corresponding to Figure 8 with the same parameters. (b) Ratio of signal variance to noise variance for several components (see key) versus connectivity 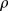. Peak synaptic conductance varies as 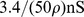 and peak IPSP size as 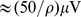. (a,b) 500 cell network simulations of 168 seconds under 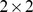 second input switching. Synaptic strength 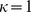. Inhibitory neurotransmitter timescale 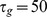 msec.

In Figure 9(a) we show PSTH variance (dashed lines) and PSTH noise (solid lines) versus component number for the three different network simulations of different connectivity investigated in Figure 8. At intermediate connectivity, 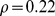, PSTH noise (solid red) is significantly suppressed below PSTH variance (dashed red) up to about component 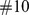. On the other hand at high connectivity 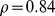 (green) only the first three components show suppressed noise while at low connectivity, 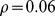, (black) little noise suppression is evident for any components except the first.

To quantify noise suppression in Figure 9(b) we show the ratio of PSTH variance to noise variance versus connectivity for several components. At high connectivity this quantity is large for only the first three components while as connectivity decreases more and more components start to show considerable noise suppression. The higher the component the greater the noise suppression in general. At connectivity around 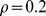, 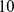 components show noise suppression. Interestingly there appears to be quite a sudden transition from high noise suppression to low noise suppression around component number 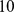. Noise suppression weakens again as connectivity decreases further however. Again the peak of noise suppression in most components occurs in the transition regime close to the striatally relevant connectivity region.

## Discussion

In this paper we investigate how a minimalistic model of a local striatal MSN network responds to variations in cortical driving.

We first illustrate using a spike raster plot and mean similarity matrix that the MSN network model can display cell assembly population dynamics locked to stimulus onset times, as previously demonstrated in [29]. We next investigate under what network conditions the reproducibility of stimulus onset locked dynamical evolution across repeat presentations of a given stimulus is maximized and for how long the reproducible patterns persist after stimulus onset. To this end we analyse how 500 cell networks respond to temporally varying cortical driving using a 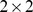 second input switching protocol. As discussed in the model section MSN networks of size 500 with connectivity around 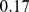 and IPSP sizes around 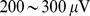 provide a reasonable representation of real local MSN network connection structure. By varying parameters individually, so that other factors are kept constant, around this striatally relevant regime we show that dynamical evolution is significantly reproducible for up to about a second after stimulus onset, but, remarkably, only at striatally relevant connection probability and IPSP size. These behaviourally relevant time scales are much longer than any represented in the model parameters. Dynamical evolution is most reproducible soon after stimulus onset and decays thereafter. Outside the striatally relevant parameter range reproducibility is much weaker for all epochs after stimulus onset.

We also investigate how stimulus distinguishability depends on IPSP size. Soon after stimulus onset the current stimulus is only weakly distinguishable from the previous one for all connection strengths. Distinguishably increases with time elapsed from stimulus onset. Most remarkably we find that the background activity (at long times after stimulus onset) generated by different stimuli shows a maximal distinguishability and this maximum occurs at striatally relevant IPSP size. In the striatally relevant parameter regime stimulus distinguishability takes about a second after stimulus onset to saturate at its maximal value.

To shed light on the origin of these optimal properties we investigate how the network generated dynamical activity of 500 cell network simulations under constant (fluctuating) excitatory drive, without input switching, depends on connectivity and connection strength. We find a transition in network generated dynamical activity around 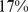 connectivity. At connectivity greater than this we find a winners-take-all like regime where some cells fire fairly regularly and the rest are quiescent. On the other hand at lower connectivities we find that most cells participate in network activity but fire in a very bursty way. We also find that the MSN network under constant (fluctuating) excitatory drive shows a connection strength dependent transition when IPSPs have size around 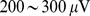, also separating a winners-take-all like regime from a regime where most cells are actively burst firing. Interestingly in both transitions the proportion of active cells shows a minimum, approximately 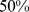, close to where the mean cell CV crosses unity. Most remarkably both transitions occur in the striatally relevant parameter range. CVs somewhat greater than unity are also commonly observed for MSN cells [13], [15], [26] and our results are thus in good agreement with observations.

To understand the network transition in more detail we investigate a simplified deterministic model of the network rate dynamics with parameters set exactly as in the full model. We are able to accurately reproduce the connectivity and connection strength dependence of network statistical quantities as well as the transition at striatally relevant parameter settings. We also numerically compute the maximal Lyapunov exponent and show that the network is marginally stable at striatally relevant parameter settings, separating a chaotic from a stable regime. In the stable regime the vast majority of network simulations show fixed point dynamics, especially at high connectivity, (see Supplemental Text S1). However at lower connectivity in the stable regime just above the transition to chaos some simulations display periodic dynamics. These interesting transitions will be the subject of future studies.

There are quantitative differences in the behaviour of the relative entropy and proportion of active cells between the rate and spiking models however. This is mainly due to the absence of dynamical effects induced by the spiking. Spiking causes noisy fluctuations around the fixed point states which reduces the relative entropy and may affect stability of attractors in the rate model. The periodic dynamical states are less likely to be observed in the full spiking network. Also transient periods of spike phase locking which may occur in the full spiking model [44] are absent in the rate model. Differences also result from approximating the firing rate dependence of the conductance based synapses by a fixed value (see Methods), and from the absence of noise in the excitatory driving.

We next ask whether stimulus onset locked reproducible dynamics is mediated by single cells or by MSN cell assemblies with coherent slowly varying rates. To investigate this we apply principal component analysis to firing rate time series generated using a long 100 msec time window. Temporal variation in principal components is generated by the coherent activity of populations of cells. We show that at high connectivity only the first three population components show strong dependence on cortical variations. The first component represents the winning set of cells while the next two only activate transiently at stimulus switches. Network dynamics appears very stable and activated components rapidly relax between two fixed point states, one for each stimulus, characterised by different stimulus dependent distributions of regularly firing and quiescent cells across the network. As connectivity decreases more and more population components display reproducible dynamics after stimulus switches, peaking at around 10 at striatally relevant connectivity. The temporal variations of these components are generated by the coherent activation and deactivation of different subpopulations of cells which inhibit and disinhibit each other. At connectivities near the transition the network successively visits different transient distributions of active and quiescent cells before eventually finding a stable distribution. As connectivity decreases further population components appear to become unstable, wandering apparently randomly without locking to stimulus onset times. Thus cortical driving interacts maximally with network generated population activity at striatally relevant connectivity.

Now we discuss how these results can be explained within the framework of dynamical systems theory. There have been many investigations of dynamical regime transitions in networks of excitatory and inhibitory neurons. Regimes of synchronous and asynchronous irregular activity as well as oscillatory regimes have been found [45]–[48]. Sompolinsky et al. [49] found a transition from a stationary phase to a chaotic phase in a network of nonlinear elements interacting via random asymmetric couplings. The random firing activity in the asynchronous regime was shown to be generated by chaos produced by the quenched random network structure. The transition from synchronous to asynchronous activity which occurs when the network balance changes from excitatory to inhibitory is accompanied by a sudden transition in ISI CV from a value close to zero to one much larger than unity. However these studies treat a network in the limit of sparse connectivity 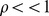 so that correlations in fluctuations in input a cell receives can be neglected. In this sparse limit the inputs to each cell from the rest of the network are described by a single common time varying firing rate. The calculations do not apply when significant correlations appear beyond those induced by this common rate. The network studied here with 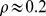 is far from this limit. Indeed we specifically investigate the dynamical switching of cell assemblies [26] which are groups of transiently strongly correlated cells. Moreover different cells have very different temporal modulations of their firing rates. However our results, in particular the fact that CV values are close to unity, so that spiking activity is Poissonian, and the fact that 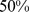 of the network is active in the transition regime, suggest that the network may be close to balanced in the transition regime.

In more recent work closely related to the present study, Buckley and Nowotny [50] investigated a bifurcation in stability in two asymmetrically connected populations of inhibitory conductance based neurons. The neurons were coupled by more biologically realistic conductance based synapses (as they are in the present work) where the synaptic current depends on postsynaptic membrane potential. This can produce effects not accounted for by current based synapses [51]–[53]. They also included a slow inhibitory neurotransmitter decay timescale as we do here. They show that near the bifurcation where the globally stable fixed point is close to losing stability the inhibitory network has optimal properties, maximizing dynamic range, and displaying slow transient dynamics after excitatory input pulses. They relate this behaviour to critical phenomenon occurring near phase transitions and show that the bifurcation results from the competition between inhibitory populations. When the fixed point is unstable the system exhibits oscillations, chaos or saturating dynamics depending on the connectivity matrix. The present work extends this study to a random inhibitory network modeling the striatal MSN network and investigates its behaviour under variation of connectivity and connection strength. Performance has been shown to be optimal in the marginally stable state known as the ‘edge of chaos’ in several studies of networks which exhibit a transition from stable to chaotic dynamics [36]–[39]. In recent work Toyoizumi and Abbott [37] determine analytically that the signal-to-noise ratio of large randomly connected networks diverges in a critical state near the edge of chaos, and the memory lifetime of the network also diverges. In fact they find performance is optimal in the chaotic regime close to the transition.

Here we offer the following rough explanatory scenario. Our simulations of the deterministic reduced rate network suggest that the phenomenon we observe here is related to ‘critical slowing down’ occuring in marginally stable weakly chaotic transient dynamics close to the edge of chaos. Indeed (at least) two factors seem relevant for the generation of complex reproducible dynamics in the present random network model under the periodic forcing of the stimulus switching. First the dynamical trajectories generated by the network dynamics should remain quite complex and high dimensional for long periods after stimulus onset. If this is not the case multiple different states in a sequence cannot be discriminated or the elapsed time represented in this random network model. Second network dynamics in the periodically forced system should be stable with period of the forcing stimulus. The stability of dynamics under periodic forcing depends of course on the stability of dynamics generated by the autonomous network in both the stimuli in the absence of forcing. However it also depends on other factors such as the period of the forcing stimulus. In general periodic driving can cause stable activity states to become chaotic and vice-versa. Indeed Rajan et al. [42] have recently shown that periodic forcing can suppress neural network generated chaotic dynamics in a frequency dependent way. They also show suppression of chaotic activity depends on the strength of the forcing.

One way in which activity in the transition regime between stable and unstable behaviour can be both complex and reproducible is due to temporally extended activity which would be transient to a stable fixed point in the unforced system. Indeed deep in the winners-take-all regime, far from the transition, network activity in the unforced system is characterised by a very stable stimulus dependent fixed point. In the periodically forced system after the excitatory input is switched the system moves to a new fixed point. The system moves rapidly between the fixed points due to their strong stability and with a highly reproducible trajectory due to the consistency of initial state across repeat stimulus presentations. Reproducibility is reflected in the strong noise suppression seen in all activated components (Figure 8(a)), but only three components are activated and only briefly. Thus dynamical evolution is highly reproducible but low dimensional and short lived. As the transition is approached from the winners-take-all regime by varying the network parameters the fixed points become less stable and the system takes longer to relax to the new fixed point after the stimulus is switched, lingering in the vicinity of the old fixed point. This dynamical slowing near the transition can generate complex transients on timescales much longer than those represented in the model parameters. Thus for extended transient periods after stimulus onset firing activity resembles the previous stimulus. Indeed in our simulations stimulus distinguishability increases with time elapsed after a stimulus switch.

On the other hand deep in the unstable regime reproducible stimulus locked dynamics does not occur even in the completely deterministic reduced rate network simulations (data not shown.) Here dynamical activity is complex and high dimensional, requiring many principal components to explain is variance, and thus can easily generate a sequence of strongly differing states. However since nearby trajectories rapidly diverge the network activity state at stimulus onset is strongly varying across repeat presentations and reproducibility is lost.

Transient activity in the unforced system may be complex and higher dimensional close to the transition due to the proximity of periodic and chaotic states and the prescence of attractor ruins. Attractor ruins are regions of phase space where attractors are weakly destabilized and close to which the flow is still very slow [54]. Indeed in the deterministic simulations of the reduced rate network we also find stable periodic states, limit cycles (torii), as well as chaotic states in the transition regime (see Supplemental Text S1), suggesting the proximity of Hopf bifurcations. In this case transients initiated after stimulus changes may decay to the new fixed point in an oscillatory fashion. Indeed the particular example of principal component time series shown Figure 8(e), in the transition regime, may indicate persistent oscillatory waves of inhibition and disinhibition between cell populations. More complex scenarios include slow switching along sequences of metastable ‘saddle-sets’ via heteroclinic channels, as has been shown to occur in asymmetrically coupled inhibitory rate networks (like the reduced rate network studied here) by Rabinovich and coworkers [55], [56] in the paradigm of winnerless competition. Here the trajectory remains in the vicinity of a metastable saddle-set for an extended period before suddenly moving off to the next one. Saddle-set states may be fixed point like, corresponding to firing rate cell assemblies, particular transient short-lived distributions of active and quiescent cells, or more complex dynamical attractor ruins. Rabinovich et al. [56] also demonstrate that this scenario can be preserved even in the presence of noise. Deco and coworkers [57], [58] have also studied switching between ‘ghost attractors’ in a critical regime near a transition to a multistable state. However besides transient activity we should also mention that in the transition regime activity generated by one stimulus may be chaotic while in the other stable fixed point thus the periodically forced activity will be stable but complex. In general we suggest the MSN network is in a marginally stable regime facilitating the generation of weakly chaotic and complex transient activity. When the network is periodically forced by the stimulus switching this activity can produce complex but stable periodic activity.

This scenario is consistent with the observation that the transition seems to occur when the network is just balanced, as discussed above. In the winners-take-all state the permanent quiescent component allows the remaining active cells to fire fairly regularly, thus reducing the mean 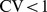, while in the chaotic switching state the transient activation of sets of cells in assemblies produces the highly bursty 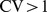 values. The transition thus occurs when 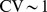. That this transition happens to occur in the striatally relevant parameter regime is non-trivial and unexpected.

Suggestions of critical dynamics can be seen in the the PSD of the higher components in the intermediate (Figure 8(d)) and low (Figure 8(f)) connectivity simulations. These display a region of rapid growth in the range 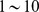 Hz which appears approximately linear in the log-log plot, so that 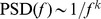, where 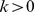. PSD profiles like these are often observed in empirical neuroscience studies [59]–[61]. For example the periodogram of spike trains of retinal ganglion cells studied in [59] (Figure 8(a)), shows a region of power-law like growth over one order of magnitude from a minimum at some intermediate frequency before saturation at low frequencies. Even though the power-law like behaviour is evident over only a fairly narrow frequency range the authors [59] claim it is evidence of fractal self-similar dynamical behaviour over this range of time scales. It should be remembered however that the PSD we calculate here are for principal components not single cells, and thus represent aggregate fluctuations of many cells. Nevertheless similar results are observed for single cells (data not shown.)

Indeed we observe (Figure 8(b,d,f)) that the slope of the power-law of the PSD of the highest components in this frequency range, 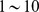 Hz, increases with decreasing connectivity. At low connectivity slopes exceed two, 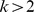, (data not shown). If power-law scaling is present on all timescales a slope of 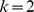 is consistent with Brownian motion. Our PSD results at low connectivity, where 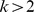, suggest that the higher components behave ‘locally’ (on short timescales) like random walks [62], so that the expected size of component fluctuations across an interval of time 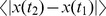 increases with the time interval 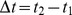 for intermediate timescales 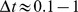 secs, before saturating at a constant level for longer time scales, as shown by the saturation of the PSD at low frequencies. This is also consistent with our observation of chaos (which generates fractal dynamical trajectories) [62], [63] in the rate dynamics at low connectivities. At high connectivity we find slopes 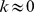 indicating a white noise like process. This is consistent with the observation of stable fixed point like rate dynamics decorated by high frequency fluctuations generated by random spike arrival times. At striatally relevant connectivities slopes 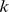 are close to 1 (data not shown) as in [59] (Figure 8(a)). Although there are various origins of 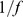 noise [61], it has been associated with criticality [64] and thus our PSD results may also be consistent with the scenario of critical slow dynamics near a transition.

Neural activity has often been modeled as a marginally stable critical process. Usually this is based on spiking activity. For example in a ‘critical branching process’ [65]–[67] spiking activity ‘just propagates’ across a network without exploding or dying, but slower rate variations can also be marginally stable [37], [50]. Since the dynamics is between a stable pattern of activity and random behavior spatiotemporal activity is highly susceptible to perturbations and the macroscopic behaviour of large cell populations can be affected by small events. Even though interactions are only local critical systems develop correlations which extend over large temporal and spatial scales compared with the scales represented in the system parameters. These characteristics make make criticality an attractive scenario to embed neural information processing.

We have shown that in the vicinity of the transition the network displays optimal properties. A variety of optimal properties have been associated with marginally stable and critical behaviour in neural systems [68], [69]. It has been suggested to optimize information transmission [70]–[73], sensitivity to sensory stimuli and dynamic range [50], [74], or memory size and computational abilities [38]. Critical and metastable dynamics [75] can also facilitate rapid adaptation to changes in processing demands [60], [76]–[80]. Self-organized critical systems include mechanisms to maintain themselves in the critical state [60], [64]. In a similar way the MSN network may remain critical by dynamically self-regulating its properties, through growth, pruning or plasticity [33], [81], [82], for example.

Chaotic balanced networks [46], [83] are thought to be responsible for the irregular bursty activity which has often been observed throughout the brain [84]–[87]. Furthermore neuronal variability is also often observed to be task dependent [84]–[91]. A sudden reduction in firing variability after stimulus onset has been observed in several recent studies [92]–[97]. Although there is no striatal study available our results of noise suppression after stimulus onset, previously reported in Ponzi and Wickens [98], are in good agreement with these studies.

Many *in-vivo* behavioural studies, in particular of reinforcement learning and temporal credit assignment tasks, show that coherent slowly varying activity in cortico-BG microcircuits is important in the encoding of movement [2], [15], [99]–[103] and the execution of learned motor programs and sequence learning [1], [104]–[114]. The quantities we chose to investigate here, the reproducibility of stimulus onset locked activity and stimulus distinguishability, are both highly relevant for such tasks. Our cortical input switching protocol, although it is the simplest conceivable, may still approximate the sudden stimulus changes which occur in such tasks. Such stimuli changes include the sudden appearance of visual cues typical in primate studies and sharp onset auditory tones in rodent maze tasks. The spatio-temporal dynamics [55], [56], [69], [115]–[120] generated by this network could be utilized in such behavioural tasks or in ‘reservoir computing’ style cognitive processing [27], [38], [39], [55], [121], [122]. In particular since network activity generates a diverse set of both stimulus and temporally specific cell responses [27], [123]–[125] it could be useful to provide fluctuations at specific times after a specific behavioural event, necessary to facilitate exploration of both sensory input and motor response [126]–[129] or simply to drive temporally delayed motor response or to control the timing of a dopamine signal in temporally delayed reinforcement learning.

In agreement with this work a variety of diverse response profiles with phasic activity peaks covering a wide spectrum of delays after task events has been observed in such tasks [8]–[10]. Jin et al. [8] found MSNs with responses so diverse that they suggested the cells could have encoded time as a population, even though the animals were performing a simple task that did not have precise-timing requirements. The authors concluded that their results could not be accounted for by a distribution of response latencies to visual inputs. They suggested additional mechanisms were needed to generate the observed response profiles, which had timescales much longer than the visual response range, and that the brain may intrinsically have properties for forming the basis of temporal computations even when not needed by the task.

Recently Adler et al. [13] addressed whether MSN activity is internally generated within the striatum, or whether it is driven by cortex. They found MSN cell assemblies which were activated at different latencies after cue presentation in a behavioural task. The cell assemblies were not differentiated by intrinsic MSN cell properties, nor were such assemblies found for the other striatal cell types investigated. For example the tonically active interneurons activated rapidly on cue presentation without distinct clustering, while clusters of globus palidus cells also did not show different activation latencies. The authors suggested that the sequential MSN cell assembly activations could be a result of MSN network dynamics. Here we show that such activity could in principle be generated internally by the MSN network and furthermore that cortical input may be transformed by the striatum in a complex way.

The principal components representing population activities which we study here might be functionally and behaviourally relevant themselves and their activity might be indirectly observed through local field potential activity. Component activities are weighted summations of MSN activities with both positive and negative coefficients, a computation which could perhaps be performed by inhibition and disinhibition of globus palidus targets. In theory their activation could be utilised by the animal to represent stimulus onset and offset in behavioural tasks. Waves of cell assemblies could be used by an animal to mark time epochs from salient stimulus switches as well as the directionality of the switching and modulate central pattern generators by inhibition and disinhibition of downstream targets.

In the simulations reported here we use excitatory drive with a fairly broad distribution of excitations across cells, quantified by the input specificity parameter 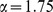 (see Model.) In previous work, Ponzi and Wickens [26], we showed that when excitatory drive was weakly above firing threshold and took a more equal value across cells network dynamical activity showed a smoother dependence on connectivity, with episodic bursting activity decreasing with increasing connectivity. In contrast here we find a more sudden transition in network activity. Indeed we find (data not shown) that decreasing the stimulus specificity by increasing 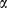 reduces the stability of the winners-take-all state, generating higher 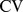 values, closer to 1 in this regime while in the unstable regime 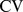 values are unchanged or slightly decreased. The regularly firing and permanently suppressed cell populations occur more strongly when input specificity is increased (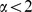) [29] and only then does stimulus onset locked dynamics occur in the transition regime. At higher 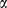 activity is chaotic throughout the whole connectivity range. It should also be noted that increasing the average excitatory input strength (as opposed to its distribution across cells) can suppress the chaotic side above a critical value of the input strength, as shown in [29] and by Rajan et al. [42].

Indeed experimental studies show that learning to perform procedural tasks alters neural firing patterns in the sensorimotor striatum as behaviour becomes more stereotyped [112], the task related activity of some cells enhanced and others suppressed. Input specificity and strength could be under the control of neuromodulators affecting corticostriatal transmission. As learning proceeds the MSN network generated fluctuations may be reduced and the network ‘locked’ when excitatory input strength or specificity is increased. Levi et al. [130] have also modeled how changes in input to a network can switch between regular and random motor behaviour. The proximity of the striatum to the transition may facilitate the rapid switching between exploratory and exploitative modes.

The model may be tested experimentally by comparing single cell MSN responses to manipulations of cortical input induced by stimulus changes in behavioural tasks, or by optogenetic activation in slices, before and after exclusively blocking lateral inhibition between MSNs. If blocking lateral inhibition changes MSN time courses then the model is supported. Due to noise suppression we also predict a sudden decrease in MSN Fano factors after stimulus onset which may be removed by blocking inhibition. However stimulus onset locked dynamics only occurs for stimuli of sufficient specificity 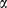 [29], thus experimental confirmation may only be possible with sufficiently salient stimuli, either due to ‘surprisingness’ or reward association.

There are many good models of the striatum, for example [131]–[136]. Like all neural models the model presented here simplifies much. However this local MSN network model is not intended to be a complete model of the striatum which in reality includes several types of interneurons, spatially organized different MSN types, feedback from extra-striatal areas etc. In general synaptic input to MSNs can be divided into two types, feedback and feedforward. Although we do not do this explicitly here purely feedforward input could simply be considered to be included in the feedforward excitatory cortical driving. However probably all synaptic input is ultimately feedback if long enough timescales are considered. For example the thalamo cortical loops will provide feedback to MSNs on long timescales in certain circumstances. Other nuclei of the BG may be involved in feedback interactions with MSNs on intermediate timescales. Striatal interneurons such as the FSIs can also provide feedback, although their main role seems to be a more global feedforward regulation of cortical driving. In this sense the feedforward component of FSI activity can also be considered an offset in the excitatory driving current. However we do not claim that these factors will not alter MSN network activity. Rather this work is an attempt to understand the recurrent dynamical properties of the local MSN network itself as a first step to understand how other striatal cell types may modulate its basic behaviour. McCarthy et al. [137] also recently investigated the MSN network in isolation. The use of a different MSN cell model leading to different types of network behaviour illustrates how useful minimal models may be for making different predictions about (some aspects of) network dynamics which can be easily traced back to differences in the elements of the model. Here we have demonstrated that the MSN network itself can show behaviour as complex as observed in experimental studies. We hope the appreciation of this complex behaviour may afford insight into the role interneurons and other striatal complexities perform in its dynamical control.

## Methods

### Model

The network is composed of model MSNs with parameters set so they are in the vicinity of a bifurcation from a stable fixed point to spiking limit cycle dynamical behaviour [26], [44]. This models the dynamics in the UP state when the cells are all receiving excitatory drive to firing threshold levels of depolarization. To describe the cells we use the 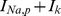 model described in [138] which is two-dimensional and given by,
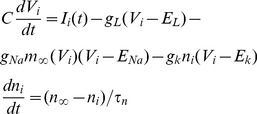
(1)having leak current 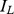, persistent 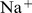 current 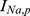 with instantaneous activation kinetic and a relatively slower persistent 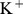 current 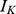. 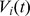 is the membrane potential of the 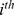 cell, 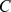 the membrane capacitance, 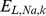 are the channel reversal potentials and 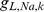 are the maximal conductances. 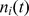 is 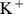 channel activation variable of the 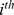 cell. The steady state activation curves 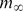 and 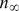 are both described by, 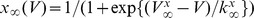 where 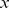 denotes 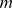 or 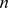 and 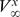 and 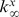 are fixed parameters. 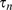 is the fixed timescale of the 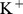 activation variable. The term 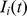 is the input current to the 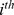 cell.

All the parameters are set as in [138] so that the cell is the vicinity of a *saddle-node on invariant circle* (SNIC) bifurcation, characterising a Type 1 neuron model. As the current 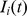 in Eq.1 increases through the bifurcation point a limit cycle having zero frequency is formed [138], whose frequency increases slowly with increasing current. This is an appropriate model to use for an MSN in the UP state, since its dynamics are in qualitative agreement with several aspects of MSN firing. Firstly the SNIC bifurcation allows firing at arbitrarily low frequencies [138] which is important since MSNs are known to fire with very low frequencies [139]. Secondly MSNs do not show subthreshold oscillations [140], [141] under normal circumstances (but see [137]). Finally the SNIC bifurcation does not allow bistability between a spiking state and a quiescent state [138] in agreement with studies of MSNs [140]–[142]. However detailed channel properties such as the interaction of L-type 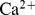 and slowly-inactivating 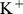 channels in the MSN have not been included in the model.

The input current 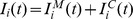 in Eq.1 is composed of two parts. Component 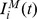 is the inhibitory feedback term which comes from the recurrent collaterals of the MSN inhibitory network and component 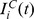 represents the current from excitatory feedforward sources, the cortex and the thalamus (see below). In the real striatum another component of the input current would come from other cells such as fast spiking interneurons (FSIs) which could be both feedforward or feedback. This component of the input current is not included in the present model, although its feedforward component can be considered simply as a constant offset term to be included in the feedforward driving 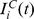. In this paper we investigate the effect MSN spiking has on other MSNs in the local MSN recurrent network to understand the dynamic feedback properties of the MSN network itself. We do not consider secondary feedback effects MSNs may have on each other via other more complex secondary pathways such as via other cell types in the striatum or via cells in other nuclei such as the Globus Pallidus, dopaminergic systems or the cortex etc.

We describe the inhibitory MSN component 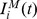 first. The inhibitory current to an MSN cell is provided by the GABAergic collaterals of the striatal MSN network. Our representation of the MSN network is constrained by the experimentally determined biological facts. These concern : (i) the network structure which determines the typical quantity of synapses an MSN makes with other MSNs, the distribution of this quantity across MSNs and the local connection probability which determines the amount of ‘recurrency’ within the local network of an MSN; (ii) the proportion of MSN cells driven by excitatory cortical and thalamic input to a state where they would be actively firing in the absence of inhibition from within the striatum; (iii) the synaptic strengths which determine the peak IPSP size elicited by a presynaptic spike on a postsynaptic cell and their distribution across network connections; (iv) the approximate time scale of decay of an IPSP back to the resting membrane potential. In this paper we vary these different parameters individually around their approximate experimentally determined values while keeping other parameters fixed to assess their individual influence on MSN network dynamics and to show that the experimentally determined values represent a rather special and unusual network configuration. In the following we explain how these experimentally determined facts are represented in the model.

The MSN network synapses are described by Rall-type synapses [143] and the input current is given by, 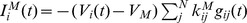. The input current to a postsynaptic neuron 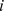 is summed over all inhibitory presynaptic neurons 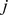 where 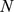 is the number of cells in the network simulation and 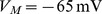 is the synaptic reversal potential. 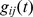 is the quantity of neurotransmitter bound to postsynaptic cell 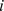 emitted from presynaptic cell 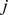. It is given through, 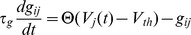. Here 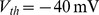 is a threshold, 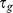 is a timescale (see below) and 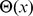 is the Heaviside function. Since the initial value of the neurotransmitter 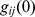 decays exponentially with timescale 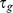, then 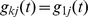 for all 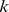 at times 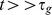 and we only need to keep track of a single 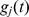 for each cell 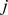. The inhibitory current into postsynaptic cell 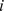 is then,
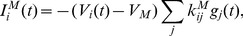
(2)and 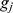 is simply an exponentially weighted moving average of cell 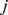 firing, given by,
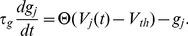
(3)Time series of 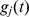 are shown in Supplemental Information, as can be seen 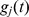 fluctuates around the cell 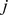 firing rate.

The representation of the MSN network is determined by the synaptic strengths 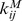 in Eq.2. These determine the peak synaptic conductance generated by a single spike which is given by 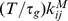 where 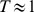 msec is the time the membrane potential exceeds 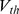 during a spike. They are given by,
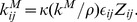
(4)Here 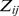 is a parameter which takes the value 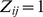 if cells 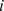 and 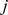 are connected and zero otherwise. 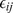 is another connection specific parameter which allows us to set individual connection strengths between connected cells 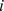 and 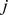. 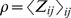 is the network connection probability. 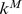 is a fixed peak conductance parameter and 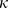 is an overall connection strength scale parameter (see below).

(i) Network structure. This is determined by 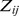. In general the connection probability between any two MSNs depends on the size of the overlap of the dendritic arborization of one cell with the axonal arborization of the other cell [34], [144]–[147]. Typical arborization diameters are around 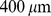, while MSN density is around 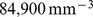 and MSN synaptic density around 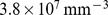 [147]. Thus within the dendritic arborization of a single MSN there are approximately 2843 other MSN of which about 448 are likely to contact (and be contacted by) the given MSN. Cells separated by larger distances than the typical arborization size have very low or zero probability to be connected. In this paper we do not consider effects arising through the long-range spatial distribution of cells across the striatum, which may for example produce complex travelling spatial temporal patterns etc, but investigate the cell assembly dynamics of a *local* randomly connected neighbourhood without a spatial dimension. Indeed this approximation is perfectly valid since the 448 connections a cell makes appear to be established randomly within a local neighbourhood without preferential attachment to cells already strongly connected to others for example.

Knowledge of the mean size of an IPSP generated on a postsynaptic cell by a presynaptic cell together with the expected quantity of active inhibitory inputs gives us the expected level of total inhibition on a cell. In our network simulations we want to respect this level of total inhibition and thus use the approximately correct IPSP size (see below) and approximately the correct number of active synapses. In our random network simulations we can respect the figure of 450 connections by choosing a network of size 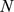 with connection probability 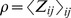 so that 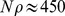. Of course this constraint can be satisfied for any network of size 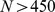 by appropriate choice of 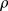. However it is clear that the connection probability 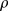 itself is an independent and important quantity, since it controls other properties such as the network ‘recurrency’. For example the quantity of inputs a pair of cells receives in common from other cells is given by 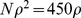 and the ratio of common inputs to all inputs a cell receives is simply 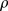. In this paper we are investigating cell assembly dynamics where cells receive strongly correlated inputs. It can thus be expected that 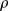 is an important parameter in this investigation. If the network size 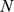 is too large the connection probability 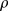 will be unrealistically small.

How do we make a striatally relevant choice for 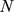 and thus 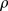
? The striatum is divided into macroscopic compartments called striosomes with width about the same as a typical dendritic arborization, 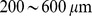 [148], [149]. It is known that the majority of MSNs have dendritic arborizations [148], [149] and axonal arborizations [149] which remain within their striosome of origin and thus this region can form a local recurrent network. Thus we choose a local network the size of a typical dendritic arborization with around 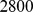 cells. A striatally appropriate value for the connectivity 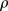 is then around 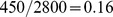


Since MSN network structure within a striosome does not indicate anything other than a random process of connection growth we connect pairs of cells randomly with probability 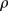 which generates networks with binomial degree distributions. There are no self-connections, 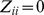.

(ii) However in this calculation there is a factor we have not yet included. This arises from the fact that during the course of any particular type of behaviour only a small proportion of MSNs are ever excited to levels above firing threshold by cortical or thalamic excitation. Such cells which can never fire can be left out of simulations altogether. Based on studies [142] we suppose that only about 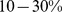 of MSNs are cortically excited to firing threshold [142] during the task period and accordingly perform simulations of 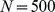 MSNs, while the remaining 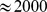 cells are considered silent. Thus at connectivity 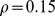 each cell receives about 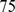 connections from other MSNs which are cortically excited during the same period and another 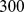 connections from cells not cortically excited across this period, which are left out of the simulation.

(iii) Connection strength and IPSP size. Synaptic conductance and IPSP size are controlled by the factor 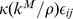 in Eq.4.

The factor 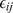 in Eq.4 is a uniform quenched random variable drawn from the interval 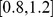 independent in 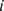 and 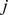, so that the expectation 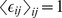, which produces a more realistic random distribution of connection strengths. Importantly this implies that even reciprocally connected cells have asymmetric inhibition, 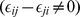, as is the case in reality.

IPSPs generated by a spike on a postsynaptic MSN close to firing threshold tend to have peak sizes of around 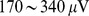 [35], [146]. Synaptic conductances in this network model are chosen so that IPSPs generated have around this size. However in this paper we investigate how variation in connectivity 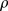 of the local neighbourhood affects network dynamics. In order to control for the total quantity of inhibition a cell feels when varying 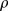 we rescale the connection strength by 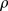, as can be seen in Eq.4, so that when the network connectivity is varied the average total inhibition on each cell is constant independent of its expected number of connections 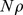. This allows us to investigate variation in local connectivity independently of the total inhibition on a cell. However it does imply that the strength of an individual synapse varies with the local connectivity 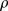 and therefore that the size of an IPSP evoked by a spike also depends on connectivity 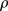. The parameter 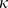 is a scale factor which allows us to vary the total inhibition on a cell independently of the connectivity 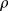. The synaptic conductance parameter 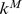 is set so that when 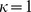 IPSPs generated by a spike on a postsynaptic cell close to firing threshold take the realistic peak size of 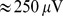 at striatally realistic connectivities of around 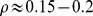 (see IPSP time courses in Supplemental Information) and at inhibitory neurotransmitter timescales 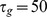 msec. It turns out that the value needed for this is 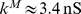. Maximal conductances at 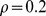 and 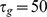 are then 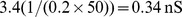. 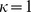 throughout this paper except where otherwise stated.

(iv) IPSP time course. In our network model we intend to reproduce the time course of recovery of the membrane potential to firing threshold after a spike from another MSN. This, together with the peak IPSP size and quantity of cortically excited incoming synapses, controls the total inhibition a postsynaptic cell receives from presynaptic spiking. This is controlled by the dynamics of the inhibitory neurotransmitter 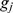. The timescale of inhibitory neurotransmitter decay 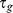 in Eq.3 has been adjusted so that the IPSP decay time scale is near that observed in experimental studies. In simulations here we generally use the value 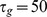 so that postsynaptically bound neurotransmitter exponentially decays to half its value in time 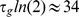 msec. This choice was motivated by Janssen et al. [30] which shows a time course of MSN IPSP with a half life of recovery of about 30–40 msec. However since a fairly large range of values has been found in various studies depending on experimental conditions as well as on cell type (D1 or D2), and also dependent on facilitating and depressing properties [31]–[35] we also investigate network dynamical properties when 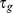 is reduced by 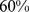 to 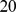 so that the half-life is 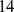 msec [31].

When changing the timescale 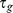 we do not change other quantities such as the synaptic strengths 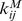. Indeed when investigating the effects of changing the timescale we need to conserve the total quantity of neurotransmitter 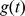 generated by a presynaptic spike, so that the level of inhibition in the network is not changed by the variation in 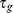. This is the quantity 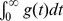. When the integral includes a single presynaptic spike this quantity is simply equal to 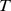 where 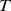 is the time period the membrane potential 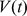 exceeds 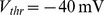 during a spike and is independent of the timescale 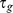. Conserving the level of inhibition does however change the peak depth of the IPSP generated by a small amount 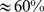 so that IPSP sizes at connectivity 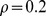 are around 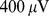 at 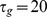 msec. This is shown in the Supplemental Information where time courses of IPSP's generated by a single presynaptic spike on a postsynaptic cell close to firing threshold are shown for both values of 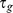. Although the quantity of inhibition is conserved, varying 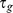 does strongly affect the size of the fluctuations in the quantity of inhibitory neurotransmitter 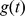 however. Time series of 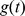 for a single cell spiking regularly are also illustrated in the supplemental information for both values of 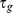. As can be seen the average levels are the same for both 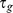 but the variance is much larger for smaller 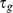. In fact both these 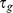 timescales are fairly large and in the network model the postsynaptic conductance can be seen to follow the exponentially decaying time average of multiple preceding presynaptic relatively high frequency spikes [44].

We model the excitatory driving 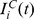 as a stochastic process. In general the excitatory component will also be given by Rall type synapses [143], [150] 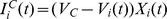 where 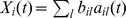. 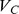 is the excitatory reversal potential, set here to the realistic value 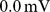. The 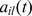 are the quantities of postsynaptically bound neurotransmitter from the 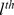 excitatory input to the 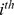 MSN cell. They are given by 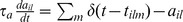 where the Dirac delta function 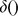 part represents a series of spikes from the 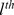 input to the 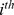 cell at times 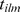 and 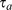 is a time scale which we set to the realistic value of 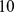 msecs. The 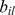 are the maximal conductance parameters from the 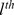 excitatory cortical or thalamic input to the 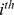 MSN cell. They are fixed in our simulations reported here, although in reality they may vary with short term facilitation and suppression as well as by 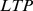 and 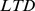.

We assume the input spikes 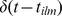 follow independent Poisson process with time varying rates 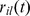. This is a simple and compact way to describe the input activity in the simulation and is not supposed to be necessarily perfectly biologically realistic. This model is an attempt to understand how the MSN network behaves under the simplest assumptions, which do not include correlation between inputs. The contribution provided by many such independent Poisson spiking processes can be replaced by a term given by the mean rate plus a fluctuation proportional to the standard deviation, assuming spikes are independent across 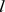 [29], [48]. Therefore we calculate 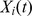 using,
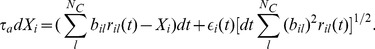
(5)


MSN cells are each contacted by around 10000 cortical and thalamic cells and we therefore set 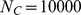 in Eq.5. These excitatory inputs 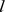 are considered to be non-overlapping between the MSN cells 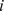. Indeed while a given corticostriatal axon will often be providing input to a substantial number of cells (about 800 on average) within the volume of the arborization zone of a typical corticostriatal axon there are about 68,000 striatal spiny neurons, making the average common input about 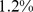 or less [151]. Our assumption of zero common input is not, however, supposed to be a statement of biological fact. We wish to investigate how correlated activity arises from local interactions among MSNs, rather than via common input.

Here we investigate how the MSN network model responds to the simplest kind of temporally varying cortical input. This is just a sequence of different stimuli, such as might occur in a visual attentional task where a shape suddenly changes colour, for example. To model this we simply change all the cortical input rates 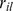 suddenly, hold them fixed for a period of time, then change them again suddenly. Each given set of rates 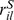, held fixed for a period of time, is denoted a stimulus, 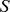. In the simulations reported here we generate two stimuli 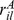 and 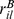 which are then applied alternately and repeatedly for two seconds each.

For each stimulus 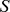 the 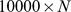 input rates 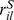, where 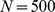 is the number of MSN cells, are drawn independently from a fixed distribution, a normalized Pareto distribution, 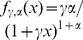, with tail parameter 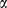 and expectation 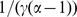. The normalized Pareto distribution with power-law tail parameter 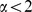 is chosen so that even though there are many, 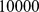, inputs to each cell the mean input strength can still show reasonably sized fluctuations across cells. Sums of power-law distributed variables (with finite variance, 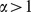) do still converge to the Gaussian distribution, but the convergence rate is very slow. If instead the 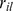 are chosen from a narrow distribution, for example the Gaussian, when many inputs are averaged all the cells will have approximately the same input strength and stimulus specificity will not be generated. Variation of the tail parameter 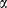 controls the size in fluctuations in input strength across cells and therefore the amount of input specificity. When 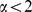 stimuli will have specificity which increases as 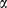 decreases towards unity. If 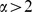 stimuli will only be very weakly discriminable since input strength 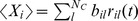 increases with 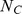 while the fluctuations in 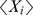 across cells 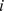 increase only with 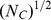.

We have chosen to use the Pareto distribution as a device to produce a large variation in excitation strength across MSN cells as only the simplest of several possibilities. There are others which may be biologically plausible, for example correlation in inputs to a single cell [12]. However since we do not consider correlations in input across different MSNs which method is chosen is not central to the modeling described here.

In this paper we do not vary the parameters of the Pareto distribution 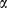 and 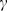 and set 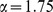 in all simulations. 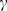 is set so that the input rates 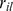 have expectation 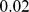 spikes per msec (due to the Pareto distribution the fluctuations across cells around this mean value will be significant). We choose all the channel parameters 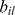 independently from a uniform distribution on 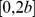. The parameter 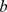 is set so the expectation of 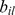 is 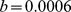. These parameters result in a mean input current of 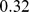 nA with standard deviation of temporal fluctuations in input current 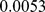 nA. If a cell 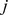 has a mean input current below the firing threshold 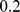 nA its 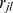 and 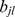 are redrawn until otherwise. Thus all cells are driven above firing threshold by the cortical excitation.

All simulations of the spiking network model were carried out with the stochastic weak second order Runge-Kutta integrator described in [152] with integration time step 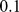 msec. All simulations are of length 180 seconds with an initial 12 second transient discarded from analysis.

### Clustering algorithm

Here we explain how the k-means algorithm is used in this paper. The number of clusters 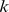 is chosen to be 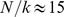 where 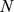 is the number of cells used in the simulation. The cross correlation matrix of cells' firing rates is calculated based on a 100 msec moving window. Each cell 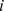 has a vector of cross-correlation coefficients 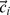. Each cluster centroid's initial location 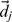, 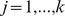 is chosen randomly as one on the cells' vector of cross-correlation coefficients 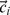. All cells 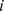 are assigned to be in the cluster 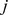 whose centroid is nearest to their cross-correlation coefficient vector, 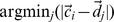 where 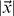 is length of 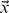. Any empty clusters are removed. New cluster centroid vectors are calculated as the mean vector of cells assigned to the cluster 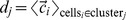. The process is repeated until there are no cells which change their assigned clusters. Notice the final amount of clusters may be (and usually is) less than the original 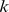. In Figure 1(a) all presentations of one of the two stimuli (here 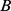) are combined into a single time series, and clusters calculated on all cells which fire at least one spike during that stimulus.

### Similarity matrices

An informative way to visualize at the time series is using firing rate similarity matrices, 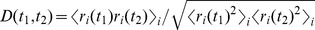 where 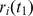 is the firing rate of cell 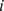 in a 100 msec time bin centered on 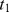 and in this expression 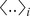 denotes average over all active cells 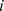 (those that fire at least one spike during the 168 second time series). This is just the overlap, taking values between 0 and 1, of the vectors of firing rates at two different times. Figure 1(b) shows a 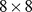 second *mean* similarity matrix, 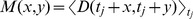 where 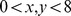 and 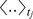 denotes averaging over 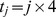 seconds, 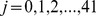, through the whole 168 second time series. That is an eight second segment is moved through the similarity matrix in steps of four seconds to create an average similarity with periodicity of the stimulation period.

Mean similarity profiles 
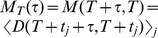
, where 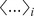 denotes averaging over all 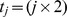 seconds, 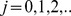, shown in Figure 2(a) are obtained by calculating the similarity between the firing state for a 100 msec time series segment centered on a reference time 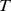 msec after the onset of a stimulus and later 100 msec time series segments at time lags of 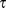 msecs (in increments of 10 msec) and averaging over all stimuli presentations. Time lags 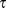 extend for 5 seconds, i.e until towards the end of the next presentation of the current stimulus. (This is the profile along a horizontal (or vertical) slice from the point 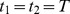 through a mean similarity matrix like the one in Figure 1(b))

### Principal component analysis

Components are generated from the correlation matrix of firing rates of all 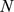 active cells (those which fire at least one spike in the observation period) based on a 100 msec time window. The principal components labeled 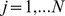 are eigenvectors such that their time series 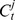 generated by the projections of network activity onto the component eigenvectors, 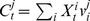 where 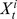 is the spike count of the 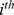 cell at time 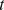 and 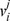 is the 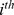 entry of the 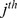 eigenvector, are not correlated with each other. Components are ordered by their associated eigenvalues, largest first, which are the variances of the principal component time series 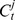.

### PSTH variance and noise variances

To calculate these quantities first calculate the 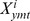, the spike counts in 100 msec windows centered on 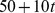 msec after the onset of the 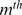 presentation of stimulus 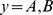 for all active cells 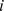. Then calculate the corresponding component time series, 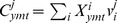, where 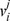 is the 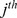 eigenvector (see above). The component 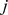 PSTH is then 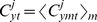 where the expectation 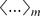 is taken over all stimulus 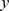 presentations 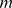. Noise variance of component 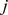 is then defined through the fluctuations around the PSTH, 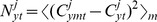. This quantity is then averaged over both stimuli 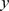 and the appropriate time 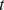 range, 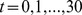, to yield the noise variance for component 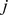, 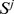. On the other hand the PSTH variance of component 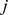 is defined through the fluctuations around the mean activity across the period that is 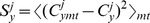 where 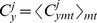 and the expectations 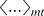 are now taken over all stimulus 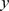 presentations 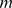 and the appropriate times 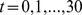. This quantity 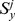 is then averaged over both stimuli 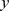 to yield the PSTH variance for component 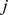, 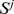.

### Relative entropy

The relative entropy (Kullback-Liebler divergence) on two normalized distributions 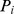 and 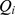 is defined as 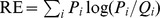. Here 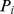 is the binned distribution of 100 msec spike counts (incremented in 10 msec steps) for a single cell. To calculate this we first find the maximum 100 msec spike count and minimum 100 msec spike count among all 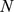 active cells in a simulation (those that fire at least one spike during the period). Then we divide this range into 500 bins 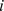 and calculate the distribution 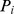 for a single cell using these bins. 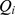 on the other hand is the distribution of 100 msec spike counts for all 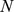 active cells in a simulation combined, using the same bins. We calculate 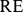 for all 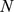 active cells in a simulation (those which fire at least one spike during the 168 second time series) and average the results to give 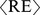. If 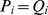 for all bins 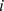 then 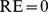. On the other hand if all cell firing rate distributions are entirely non-overlapping then when 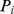 is non-zero, 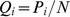 where 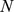 is the number of active cells, so 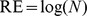. The quantity shown in Figure 5(c,d) is 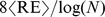 where the factor 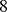 is included for convenient scaling of the figure.

### Reduced rate model

The reduced rate model is obtained from the equation for the postsynaptically bound neurotransmitters 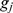, Eq.3, by replacing the Heaviside function 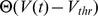 with 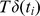. 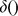 is the Dirac delta function and 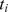 are spike times. 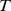 is the time period the membrane potential 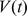 exceeds 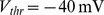 during a spike, which turns out to be 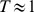 msec for this cell model. This approximation is valid here since 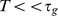. Then 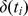 is replaced by cell 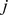 spiking probability per msec, in other words its firing rate, at time 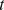, 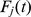 to obtain,
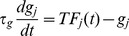
(6)Since 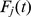 varies slowly compared to 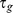, 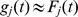 and 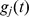 follows the firing rate 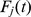. Then 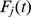 is replaced its value determined from the cell's input current to give,
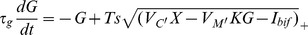
(7)where 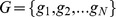 are the postsynaptically bound neurotransmitters for the 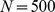 cells. 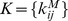 is the fixed connection matrix, determined exactly as in the simulations for the full model. 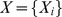, where 
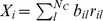
 are the expected values of the excitatory inputs determined exactly as in the full model, neglecting the noise term. 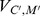 are fixed scalar parameters accounting for the conductance based synapses. These are set as the difference between the resting potentials and reversal potentials, 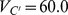 mV for excitatory synapses and 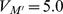 mV for inhibitory synapses. 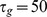 msecs as in the full simulations. The function 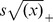, (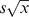 for 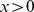 and zero otherwise), is the dependence of firing rate on input current for Type 1 cells and the parameter 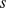 is estimated from the current versus firing rate plot for these cells to be 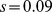. 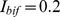 nA is the current at the firing threshold.

Statistical quantities are calculated using procedures analogous to those of the full spiking model. The proportion of active cells is calculated from the number of quiescent cells - those whose firing rate does not exceed a small value in the simulation period. A similar procedure is used to calculate the relative entropy for the reduced rate model (see above). The rates of all cells are sampled every time increment of the numerical integrator to generate the appropriate distributions. The minimum bin rate is set to zero and the maximum bin rate slightly greater than the observed maximum rate in the simulation. Quiescent cells, whose rates never exceed a small value, are not included in the relative entropy calculation. The maximal Lyapunov exponent is calculated in the standard way, as described in [29]. Numerical integrations of the deterministic system are performed using a fourth order Runge-Kutta for 110 secs and a transient of of 100 secs is discarded from the analysis. The integration time step is 1 msec, except for Lyapunov exponent calculations when it is set as 0.1 msec.

## Supporting Information

Figure S1
**Time series examples from 500 cell network simulations.** Cell raster plot time series segment and mean 8 second similarity matrix 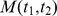 averaged across the whole 168 time series, including 42 presentations of each stimulus for 500 cell network simulations. Connection strength 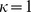 and connectivity: (a,b) 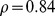 corresponding to main paper Figure 8(a,b), (c,d) 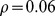 corresponding to main paper Figure 8(c,d). (a,c) Time series with all active cells shown. 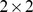 second input switching stimuli 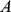 and 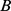 are indicated on bottom axis. Cells are grouped and coloured by k-means clusters with 30 clusters applied to only stimulus 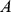. (b,d) Corresponding mean similarity matrices with colours shown in key.(TIF)

Figure S2
**IPSP and inhibitory neurotransmitter dynamics.** (a) Time course of IPSPs generated on a postsynaptic cell by a presynaptic spike (black) when the postsynaptic cell is just below firing threshold for connection strength as in a 500 cell connectivity 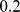 network simulation with connection strength parameter 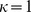 for neurotransmitter 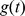 timescale 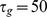 (red) and 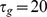 (green) (b) Time series of neurotransmitter 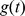 for a cell firing regularly when 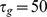 (red) and 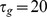 (green).(TIF)

Figure S3
**Time series examples for the reduced rate model.** Time series segments for several randomly chosen cells from 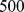 cell simulations of the deterministic reduced rate model for parameters as in Figure 6 of the main paper. Inhibitory neurotransmitter timescale 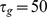 msec. Synaptic strength scale parameter 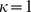. (a) Fixed point. Connectivity 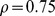 so that peak synaptic conductance is 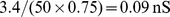. (b) Chaotic. Connectivity 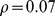 so that peak synaptic conductance is 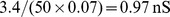. (c) Periodic. Connectivity 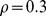 so that peak synaptic conductance is 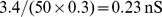.(TIF)

Figure S4
**Distribution of fixed, periodic and chaotic states in the reduced rate model.** Variance of individual cell firing rate time series averaged across all cells for many 500 cell simulations of the reduced rate model, corresponding to Figure 7 of main text. Black circles correspond to simulations with positive Lyapunov exponent. Red squares correspond to simulations with negative Lyapunov exponent. Time series had length 10 secs after discarding a 100 sec transient. Bars indicate the spread in variances across cells in the simulations. y axis log scale. All results have had a small amount added to them, 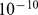, so that simulations with zero variance can be shown in the log scale. Inhibitory neurotransmitter timescale 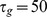 msec. (a) Connectivity 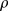 variation for synaptic strength scale parameter 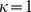 so that peak synaptic conductance varies as 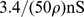. (b) Synaptic strength scale parameter 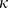 variation for connectivity 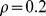. Actual peak synaptic conductance is given by 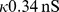.(TIF)

Figure S5
**Deterministic simulations of the spiking model show stochastic stimulus response.** (a) Mean 8 second similarity matrix 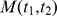 averaged across the whole 168 time series, including 42 presentations of each of the two two second stimuli for a 500 cell connectivity 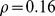, *deterministic* spiking network simulation without fluctuations in excitation. Connection strength parameter 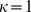, neurotransmitter timescale 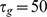 msec, so that peak synaptic conductance is 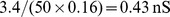. (b) Similarity matrix 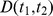 (see Materials and Methods) for a 22 second segment from the 168 second time series used to generate the mean similarity matrix in (a). (Colours shown in key.)(TIF)

Text S1
**Optimal balance of the striatal medium spiny neuron network, supplemental.** Sections: (1) Network simulations at unrealistically high and unrealistically low connectivity. (2) Effect of reduction in inhibitory neurotransmitter timescale 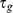 on IPSP. (3) Time series examples for reduced rate model. (4) Distribution of fixed points, periodic and chaotic states in reduced rate model. (5) Stimulus response remains stochastic in deterministic spiking network model.(PDF)
