# Supplementary figures and images for: Optimal Balance of the Striatal Medium Spiny Neuron Network
Source: PLoS Comput Biol. 2013 Apr 11;9(4):e1002954. doi: 10.1371/journal.pcbi.1002954 (PMC3623749; doi:10.1371/journal.pcbi.1002954)

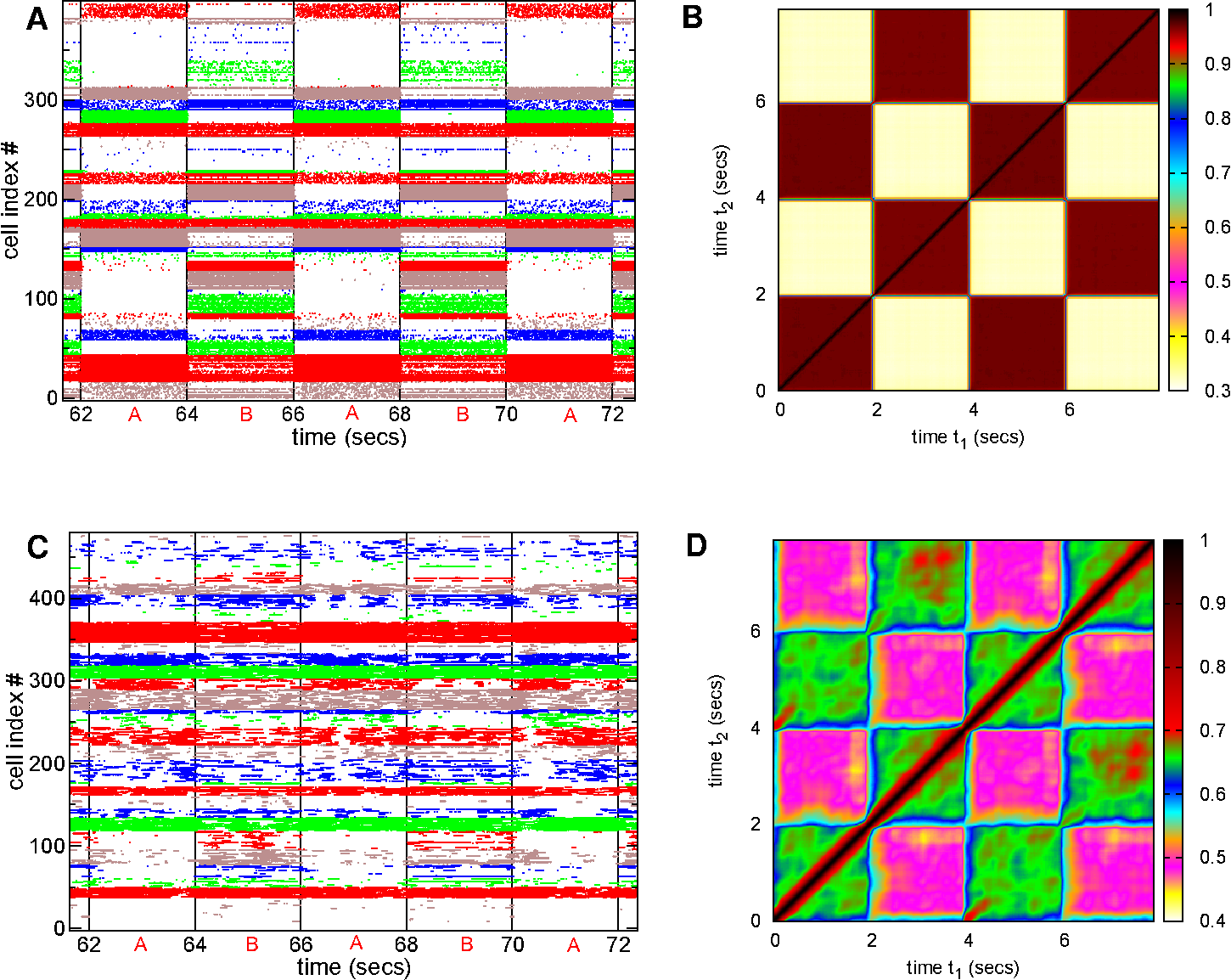

Supplement: Figure S1 — Time series examples from 500 cell network simulations. Cell raster plot time series segment and mean 8 second similarity matrix averaged across the whole 168 time series, including 42 presentations of each stimulus for 500 cell network simulations. Connection strength and connectivity: (a,b) corresponding to main paper Figure 8(a,b), (c,d) corresponding to main paper Figure 8(c,d). (a,c) Time series with all active cells shown. second input switching stimuli and are indicated on bottom axis. Cells are grouped and coloured by k-means clusters with 30 clusters applied to only stimulus . (b,d) Corresponding mean similarity matrices with colours shown in key. (TIF) [file pcbi.1002954.s001.tif]

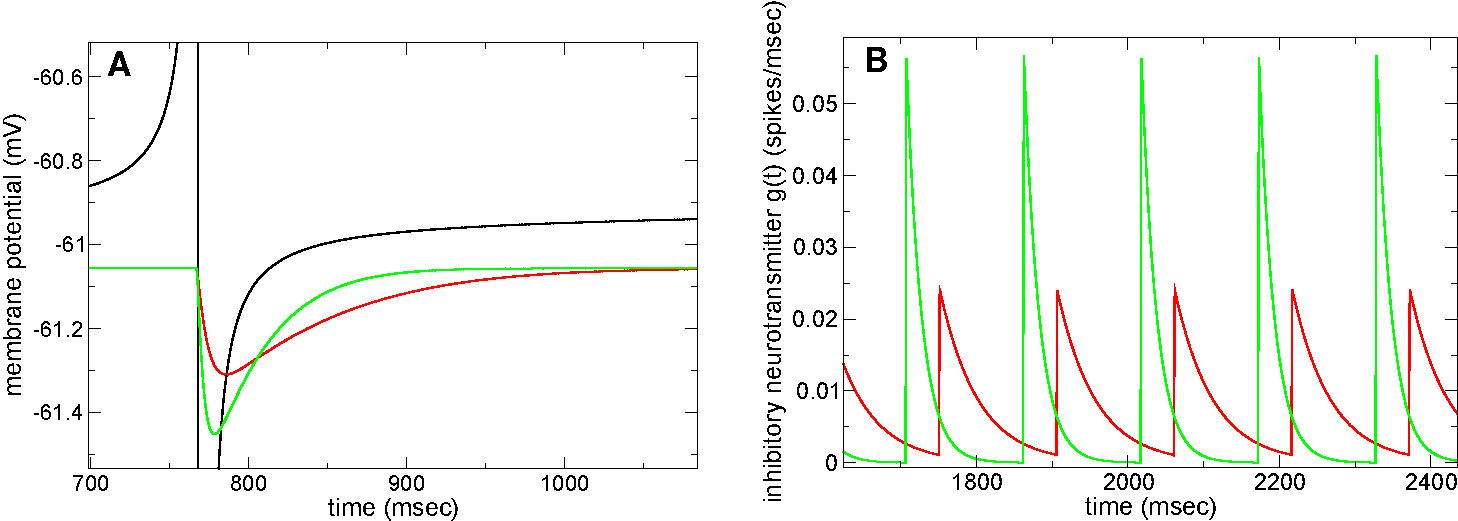

Supplement: Figure S2 — IPSP and inhibitory neurotransmitter dynamics. (a) Time course of IPSPs generated on a postsynaptic cell by a presynaptic spike (black) when the postsynaptic cell is just below firing threshold for connection strength as in a 500 cell connectivity network simulation with connection strength parameter for neurotransmitter timescale (red) and (green) (b) Time series of neurotransmitter for a cell firing regularly when (red) and (green). (TIF) [file pcbi.1002954.s002.tif]

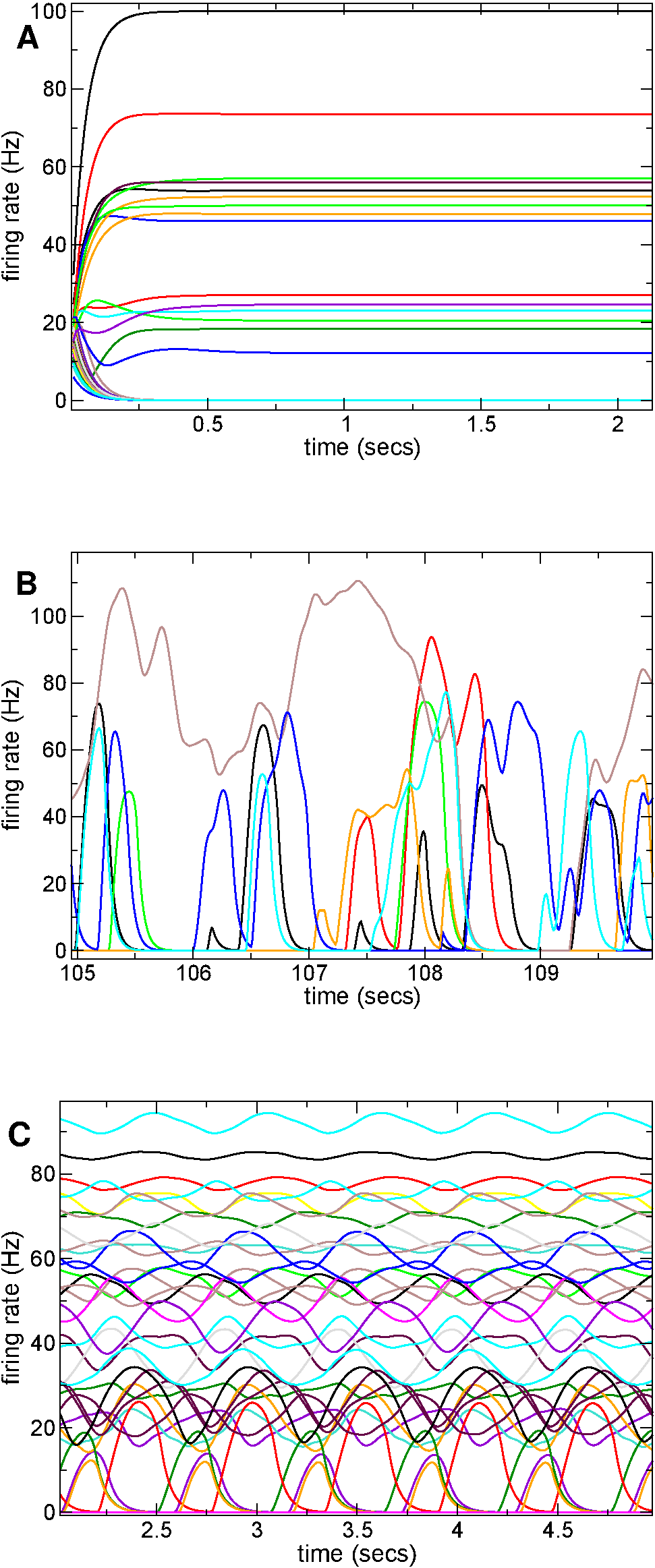

Supplement: Figure S3 — Time series examples for the reduced rate model. Time series segments for several randomly chosen cells from cell simulations of the deterministic reduced rate model for parameters as in Figure 6 of the main paper. Inhibitory neurotransmitter timescale msec. Synaptic strength scale parameter . (a) Fixed point. Connectivity so that peak synaptic conductance is . (b) Chaotic. Connectivity so that peak synaptic conductance is . (c) Periodic. Connectivity so that peak synaptic conductance is . (TIF) [file pcbi.1002954.s003.tif]

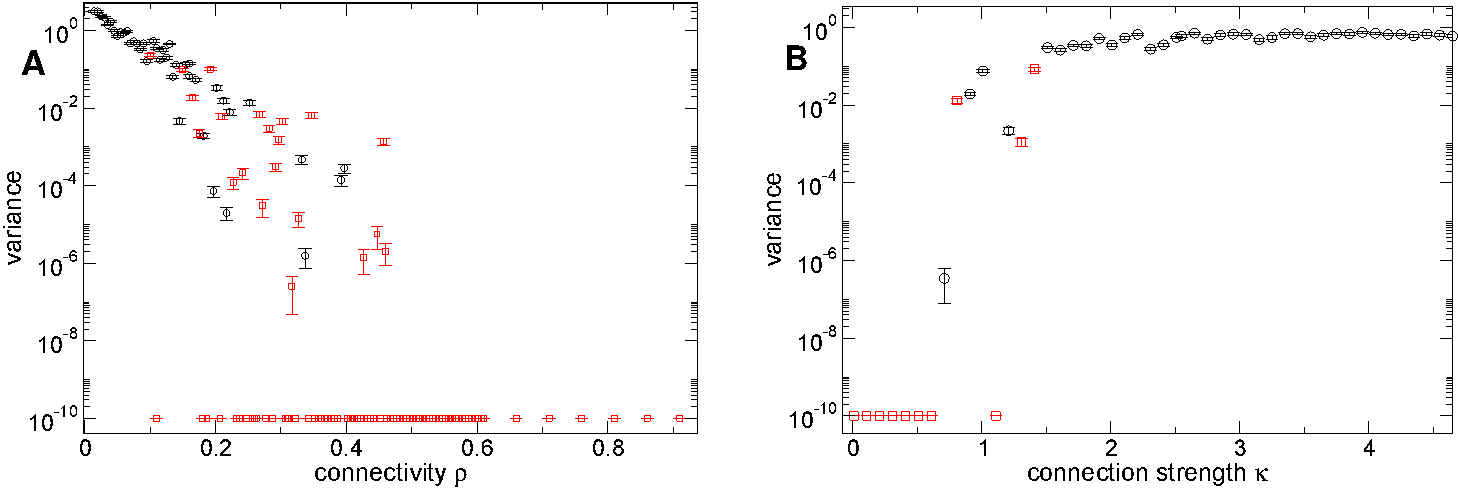

Supplement: Figure S4 — Distribution of fixed, periodic and chaotic states in the reduced rate model. Variance of individual cell firing rate time series averaged across all cells for many 500 cell simulations of the reduced rate model, corresponding to Figure 7 of main text. Black circles correspond to simulations with positive Lyapunov exponent. Red squares correspond to simulations with negative Lyapunov exponent. Time series had length 10 secs after discarding a 100 sec transient. Bars indicate the spread in variances across cells in the simulations. y axis log scale. All results have had a small amount added to them, , so that simulations with zero variance can be shown in the log scale. Inhibitory neurotransmitter timescale msec. (a) Connectivity variation for synaptic strength scale parameter so that peak synaptic conductance varies as . (b) Synaptic strength scale parameter variation for connectivity . Actual peak synaptic conductance is given by . (TIF) [file pcbi.1002954.s004.tif]

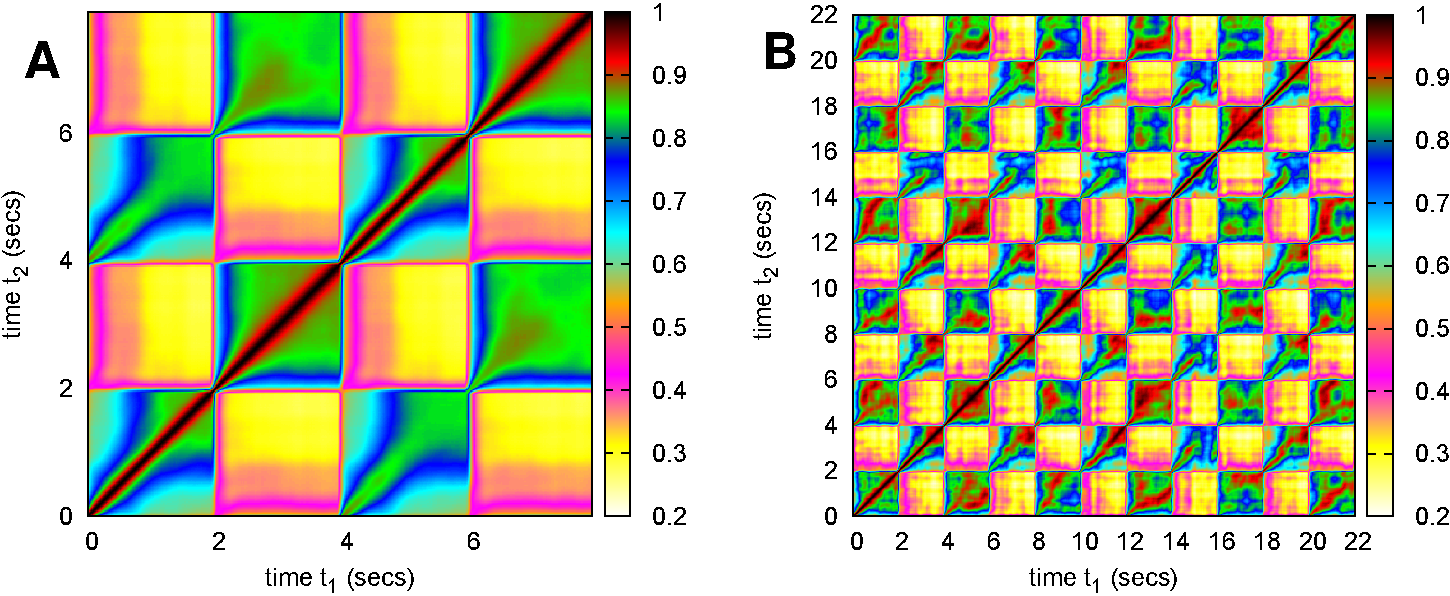

Supplement: Figure S5 — Deterministic simulations of the spiking model show stochastic stimulus response. (a) Mean 8 second similarity matrix averaged across the whole 168 time series, including 42 presentations of each of the two two second stimuli for a 500 cell connectivity , deterministic spiking network simulation without fluctuations in excitation. Connection strength parameter , neurotransmitter timescale msec, so that peak synaptic conductance is . (b) Similarity matrix (see Materials and Methods) for a 22 second segment from the 168 second time series used to generate the mean similarity matrix in (a). (Colours shown in key.) (TIF) [file pcbi.1002954.s005.tif]
