# Supplementary material for: Optimal Balance of the Striatal Medium Spiny Neuron Network
Source: PLoS Comput Biol. 2013 Apr 11;9(4):e1002954. doi: 10.1371/journal.pcbi.1002954 (PMC3623749; doi:10.1371/journal.pcbi.1002954)
Supplement: Text S1 — Optimal balance of the striatal medium spiny neuron network, supplemental. Sections: (1) Network simulations at unrealistically high and unrealistically low connectivity. (2) Effect of reduction in inhibitory neurotransmitter timescale on IPSP. (3) Time series examples for reduced rate model. (4) Distribution of fixed points, periodic and chaotic states in reduced rate model. (5) Stimulus response remains stochastic in deterministic spiking network model. (PDF) [file pcbi.1002954.s006.pdf]

Optimal Balance of the Striatal Medium Spiny Neuron Network, Supplemental

Adam Ponzi*, Jeffery R Wickens
Neurobiology Research Unit, Okinawa Institute of Science and Technology (OIST), Okinawa, Japan.
$*$ E-mail: adamp@oist.jp

Network simulations at unrealistically high and unrealistically low connectivity

Figure S1 shows cell raster plot time series segments and corresponding similarity matrices $M(t_1, t_2)$ from high and low connectivity simulations, clustered by the k-means algorithm applied to only one of the stimuli. In both simulations some cells show rates which vary depending on the stimulus however reproducible stimulus onset locked temporal patterning is absent at both high and low connectivity. At high connectivity spike processes appear Poissonian or more regular than Poissonian. At low connectivity activity is very bursty.

Effect of reduction in inhibitory neurotransmitter timescale τ_g on IPSP

In Figure S2 we illustrate the affects of variation in the inhibitory neurotransmitter $g(t)$ timescale τ_g.

Figure S2(a) contrasts the IPSPs generated in a postsynaptic cell close to firing threshold for connection strengths the same as in a connectivity $\rho = 0.2$ simulation with the synaptic strength parameter $\kappa = 1$. This means the peak synaptic conductance is $3.4/(0.2 \times 50) = 0.34$nS when $\tau_g = 50$ msec and $3.4/(0.2 \times 20) = 0.86$nS when $\tau_g = 20$. The time taken for the IPSP to decay to half its peak value is about 50 msec when $\tau_g = 50$ (red). Its peak value is about 250mV. When the timescale is reduced to $\tau_g = 20$ msec (green) the peak value is increased to about 400mV and the half life reduced to about 25 msec. However has explained in the main text the total quantity of neurotransmitter released by a presynaptic spike is the same in both cases independent of the timescale τ_g.

In Figure S2(b) we show time series of neurotransmitter $g(t)$ for a cell firing around 7 Hz for the two different values of neurotransmitter timescale τ_g. As can be seen both time series have similar mean values $\langle g \rangle \approx 0.007$ as expected however the fluctuations around this value are much larger in the shorter timescale $\tau_g = 20$ case (green).

Time series examples for reduced rate model

In Figure S3 we show examples of time series segments from the deterministic reduced rate model. In these simulations the excitatory input is fixed for the duration of the simulation without stochastic fluctuations and without input switching. The parameters are the same as in the full network simulation investigated in Figure 6 of the main paper. The low connectivity $\rho = 0.07$ example Figure S3(b) shows chaotic temporal evolution. The high connectivity $\rho = 0.75$ example Figure S3(a) illustrates the rapid approach to a fixed point with cells firing at different rates. The connectivity $\rho = 0.3$ example Figure S3(c) shows a periodic state.

Distribution of fixed points, periodic and chaotic states in reduced rate model

In Figure S4 we show how the dynamical variance of cells' firing rate time series in 500 cell simulations of the reduced rate model depends on the network parameters connectivity ρ and connection strength κ. That is for each cell i we calculate $\sigma_i^2 = \langle g_i(t)^2 \rangle_t - \langle g_i(t) \rangle_t^2$ where the expectation is taken over time t from $t = 100$ to $t = 110$ seconds in 1 msec steps. We then average σ_i^2 across all cells i for each simulation. The bars indicate the spread in σ_i^2 across cells i in each simulation.

The simulations are further divided into those with positive Lyapunov exponents indicating unstable dynamics (black circles) and those with negative Lyapunov exponents indicating stable dynamics (red squares). These figures include the same simulations as Figure 7 of the main text.

Figure S4 (a) shows the connectivity variation. The points at high connectivity $\rho > 0.5$ with zero variance (shown as variance 10^{-10}) and negative Lyapunov exponents (red squares) correspond to simulations where all cells firing rates find completely fixed levels. The points with negative Lyapunov exponents (red squares) and non-zero variances correspond to simulations where some cells have periodically varying firing rates and the rest fixed rates. The black circles correspond to chaotic states. In the transition connectivity regime $0.17 < \rho < 0.5$ there are some periodic simulations, some fixed point simulations and some chaotic simulations. Below connectivity $\rho < 0.17$ there are very few fixed points and periodic points and most simulations are chaotic.

Figure S4 (b) shows the connection strength variation. At low connection strength $\kappa < 1$ all simulations are fixed point. In the transition regime $\kappa \approx 1$ there are a few periodic simulations. Above $\kappa > 1$ all simulations are chaotic.

Stimulus response remains stochastic in deterministic spiking network model

In the main text all simulations of the spiking network model were conducted with stochastic fluctuations in the excitatory driving. Here we demonstrate that even when there are no such fluctuations and the network simulation is entirely deterministic the network still responds in a stochastic way to stimulus presentation.

Figure S5(a) shows a mean 8 second similarity matrix from a connectivity $\rho = 0.16$ deterministic 500 cell spiking network simulation under the 2×2 second input switching protocol. In this simulation stimulus A presented during periods $t = 0 \sim 2$ and $t = 4 \sim 6$ shows fairly strong stimulus onset locked reproducible dynamics for more than a second after stimulus onset. This is shown by the fact that similarity along the main diagonal in the following presentation of stimulus A, $M(4 < t_1 < 6, 0 < t_2 < 2)$ remains quite sharply peaked until about $t_1 = 5$.

Figure S5(b) shows the similarity matrix itself $D(t_1, t_2)$ (see Materials and Methods) for a 22 second segment from the time series. Stimulus A presentations occur at $t = 4n \sim 4n + 2$, for $n = 0, 1, 2, \ldots$. For example the block $D(16 < t_1 < 18, 8 < t_2 < 10)$ shows the similarity between the 5th presentation of stimulus A and the third presentation of stimulus A. As can be seen during some presentations of stimulus A the network response is strongly reproducible for long periods of up to two seconds after stimulus onset (for example $D(8 < t_1 < 10, 0 < t_2 < 2)$) while other presentations of stimulus A only show weak reproducibility (for example $D(16 < t_1 < 18, 12 < t_2 < 14)$). Thus even though the network simulation is entirely deterministic it still responds in a way which varies trial by trial. This could be an origin of error trials in behavioural tasks.
